# Descending pathways increase sensory neural response heterogeneity to facilitate decoding and behavior

**DOI:** 10.1016/j.isci.2023.107139

**Published:** 2023-06-15

**Authors:** Michael G. Metzen, Maurice J. Chacron

**Affiliations:** 1 Department of Physiology, McGill University, Montreal, QC H3G 1Y6, Canada

**Keywords:** Neuroscience, Behavioral neuroscience, Sensory neuroscience

## Abstract

The functional role of heterogeneous spiking responses of otherwise similarly tuned neurons to stimulation, which has been observed ubiquitously, remains unclear to date. Here, we demonstrate that such response heterogeneity serves a beneficial function that is used by downstream brain areas to generate behavioral responses that follows the detailed timecourse of the stimulus. Multi-unit recordings from sensory pyramidal cells within the electrosensory system of *Apteronotus leptorhynchus* were performed and revealed highly heterogeneous responses that were similar for all cell types. By comparing the coding properties of a given neural population before and after inactivation of descending pathways, we found that heterogeneities were beneficial as decoding was then more robust to the addition of noise. Taken together, our results not only reveal that descending pathways actively promote response heterogeneity within a given cell type, but also uncover a beneficial function for such heterogeneity that is used by the brain to generate behavior.

## Highlights

• Response heterogeneity within sensory neural populations serves a beneficial function
• Pharmacological inactivation of feedback strongly reduces heterogeneity
• Decoding is more robust to noise addition before feedback inactivation
• Heterogeneity is actively promoted in sensory systems to benefit coding

## Graphical abstract

**Figure:**
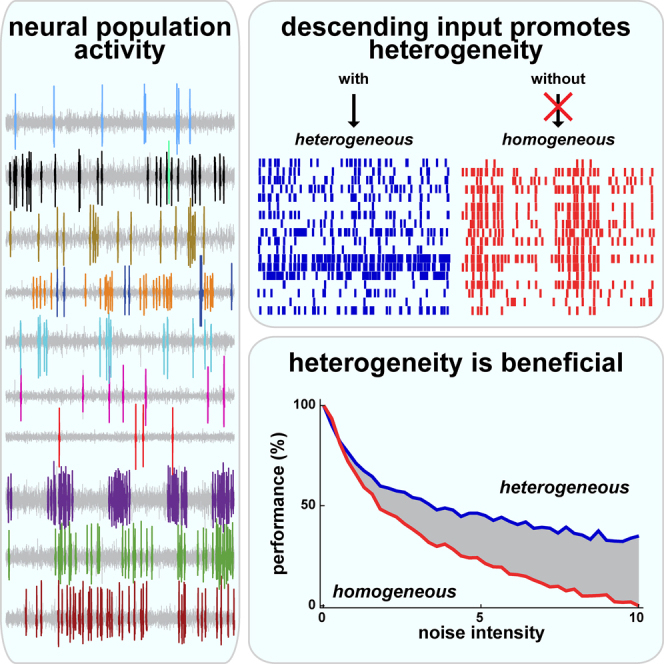

## Introduction

Understanding how neurons process incoming sensory information to give rise to perception and behavior (i.e., the neural code) remains a central problem in systems neuroscience. Although it is generally agreed that behavioral responses (e.g., perceiving the location of an object within the visual field) are determined by the activities of large neural populations,^1^^,^^2^^,^^3^^,^^4^ understanding how these activities are combined is complicated by the fact that neurons across sensory modalities and motor pathways that encode the same stimulus attribute exhibit a high degree of heterogeneity in their spiking responses to stimulation (visual,^5^^,^^6^ auditory,^7^ somatosensory,^8^ and motor^9^). Although previous studies have shown that such heterogeneity has multiple benefits such as increasing coding efficiency,^10^^,^^11^^,^^12^^,^^13^^,^^14^^,^^15^^,^^16^^,^^17^^,^^18^ coding reliability,^19^^,^^20^ as well as making learning and memory more robust,^21^^,^^22^ these are for the most part theoretical in nature and rely on comparison of mathematical models where heterogeneity can be systematically varied. Previous experimental studies have relied on comparing the responses of a single neuron to repeated stimulus presentations to those of a neural population with different response properties, such that any observed differences could not be solely attributed to differences in heterogeneity.^16^ As such, the important question of whether heterogeneity serves a beneficial function that is used by the brain toward enhancing perception and behavior remains unanswered to date.

Weakly electric fish benefit from a well-characterized anatomy and circuitry as well as behaviors consisting of changes in their self-generated electric field in response to stimulation that do not require movement,^23^^,^^24^ thereby making them an attractive model system for understanding how the activities of heterogeneous neural populations mediate behavioral responses to sensory input. The electric field is generated through the electric organ discharge (EOD) and rely on perturbations of this field to acquire information about the environment.^25^ When two conspecifics are in proximity to each another (i.e., <2 m), each fish experiences an amplitude modulation (AM) of its own EOD whose amplitude (envelope) conveys information as to the distance and relative orientation and motion between both fish.^26^^,^^27^^,^^28^^,^^29^^,^^30^ Previous studies have shown that weakly electric fish display behavioral responses such that the EOD frequency faithfully follows the detailed timecourse of envelope stimuli, indicating that information about the detailed stimulus timecourse must be encoded by sensory neurons and then transmitted to higher brain areas.^31^ This behavioral response appears to be adapted to the natural statistics of envelope stimuli and is thought to help individuals make themselves appear more dominant during social interactions.^31^ A recent study has demonstrated such envelope responses in the wild in freely moving fish, thereby making laboratory studies of such envelope behavioral responses ecologically relevant.^32^ These furthermore display habituation to repeated stimulus presentations, thereby suggesting that they are plastic and most likely change because of top-down neural signals.^31^ More recent studies have shown that changes in envelope behavioral responses during sensory adaptation require top-down input from the forebrain.^33^ Further evidence suggests that ELL pyramidal cell activity is necessary to generate the changes in EOD frequency that faithfully track the envelope stimulus’ detailed timecourse.^33^^,^^34^^,^^35^^,^^36^^,^^37^^,^^38^ Envelope stimuli are encoded by peripheral electroreceptor afferents that synapse onto pyramidal cells within the electrosensory lateral line lobe (ELL). Pyramidal cells display large heterogeneities in their spiking activities in response to stimulation^29^^,^^39^^,^^40^^,^^41^ and are the sole output neurons of the ELL projecting to higher brain structures mediating the animal’s behavioral responses.

There are two main classes of pyramidal cells, basilar and non-basilar,^42^^,^^43^ which are also referred to as ON- and OFF-type because they respond to increased EOD amplitude with excitation and inhibition, respectively.^44^^,^^45^ Both ON- and OFF-type pyramidal cells display large differences in apical dendritic morphology as well as the location of their somata within the pyramidal cell layer,^46^ and can be each separated into 3 sub-classes.^47^ Specifically, cells whose somata are located most superficially tend to display large apical dendritic trees and are referred to as superficial, whereas cells whose somata are located most deeply tend to display small apical dendrites and are referred to as deep. Finally, cells whose somata are located in between tend to display intermediate apical dendrites and are referred to as intermediate.^47^ As such, there are six ELL pyramidal cell types (ON and OFF-type superficial, intermediate, and deep). Because of a strong negative correlation between apical dendritic length and baseline (i.e., in the absence of stimulation) firing rate *in vivo*, it is possible to obtain information as to which type the recorded neuron belongs to, based on this measurement as well as from responses to stimulation. Previous studies have shown large differences in the response properties of ON- and OFF-type superficial, intermediate, and deep pyramidal cells to stimulation^48^^,^^49^^,^^50^^,^^51^^,^^52^^,^^53^ (see^40^^,^^41^ for review).

ELL pyramidal cells furthermore receive large amounts of descending input from higher brain areas including both direct and indirect projections from the nucleus praeeminentialis (nP).^54^ Previous studies based on single-unit recordings have shown multiple functions for such input including gain control,^55^ adaptive cancellation of redundant stimuli,^56^^,^^57^^,^^58^ as well as adaptively generating and optimizing responses to sensory input.^33^^,^^35^^,^^36^^,^^59^ Although the responses of single pyramidal cells within the ELL to envelope stimuli have been well-characterized,^29^^,^^33^^,^^34^^,^^35^^,^^36^^,^^41^^,^^53^^,^^60^^,^^61^^,^^62^ how they encode such stimuli at the population level is not well understood to date. Previous studies have shown that different ELL pyramidal cell classes serve different functions. For example, deep pyramidal cells provide the necessary signal to their superficial counterparts that is needed to adaptively cancel out redundant stimuli.^56^ However, the important questions as to whether: 1) descending input can influence response heterogeneity observed within a given cell class and; 2) such response heterogeneity serves a beneficial function have not been investigated to date.

Here we demonstrate that response heterogeneity is mediated by descending input and serves a beneficial function that is used by the brain to ultimately generate behavioral responses consisting of changes in EOD frequency that follow the stimulus’ detailed timecourse. We found that response heterogeneity was strongly attenuated only for ON-type low firing rate pyramidal cells following pharmacological inactivation of such input. By comparing the coding properties of the same pyramidal cell population before and after inactivation, we show that response heterogeneity serves a beneficial function in that decoders optimized to reconstruct the envelope stimulus’ detailed timecourse are more robust to noise before as compared to after feedback inactivation. Importantly, we demonstrate that this beneficial function is used by the brain to generate these envelope behavioral responses. Taken together, our results provide evidence that heterogeneity is most likely not the result of noisy developmental processes but is rather actively enhanced by neural circuits within the brain with downstream decoders tuned to take advantage of the resulting benefits to coding to generate behavior.

## Results

**Figure 1:**
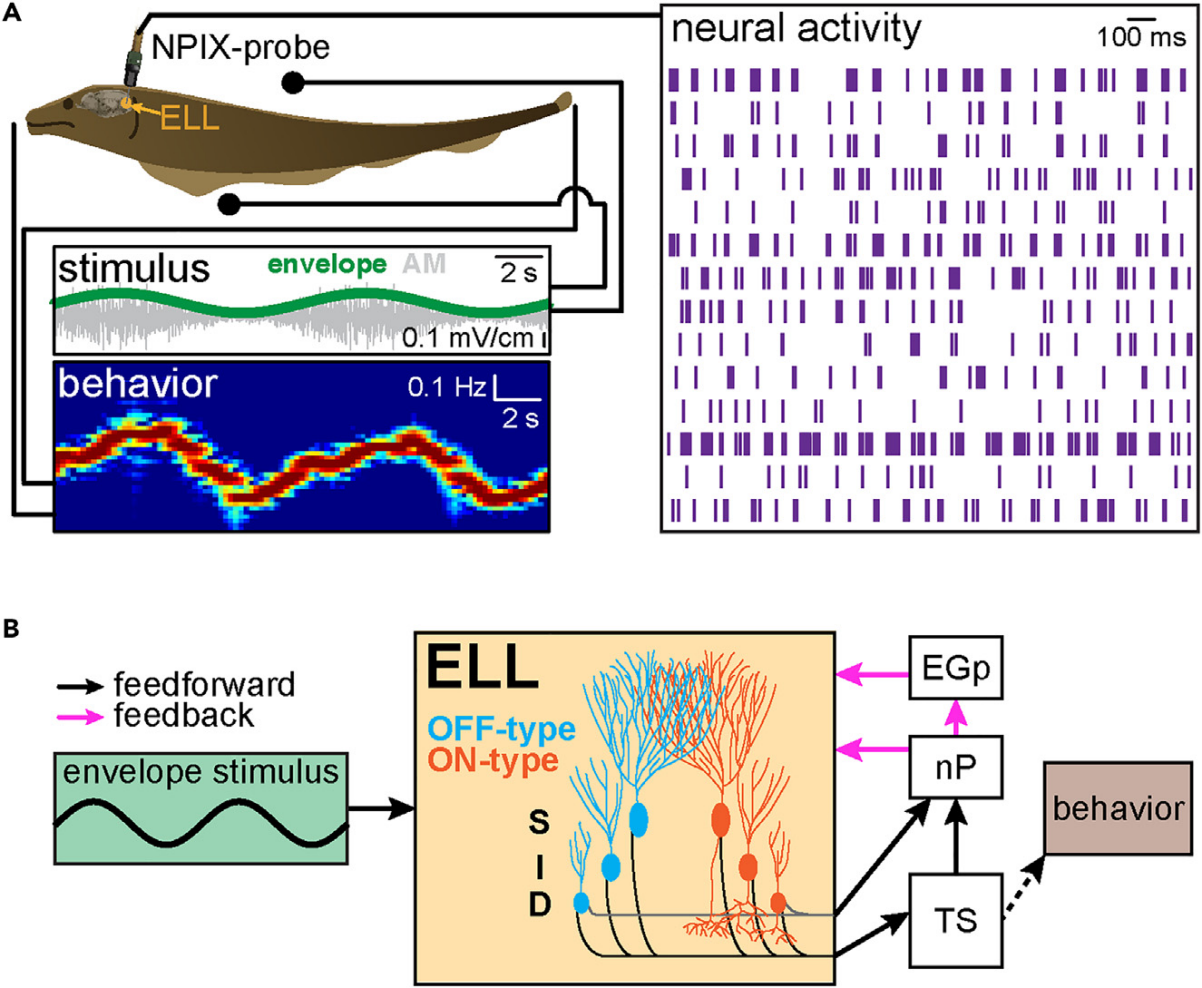
Experimental setup and relevant neural circuitry (A) The animal is placed in an otherwise empty tank and behavioral responses (EOD; bottom left) as well as neural activity (top right) are recorded simultaneously. The stimuli consisted of amplitude modulations (AMs) of the animal’s own EOD (gray, middle left) whose amplitude (i.e., the envelope, green, middle left) was modulated sinusoidally at different frequencies. The animal’s behavior (bottom left) follows the detailed timecourse of the envelope stimulus. (B) Simplified circuit diagram. The envelope stimulus is transduced by electroreceptor afferents that project to pyramidal cells within the electrosensory lateral line lobe (ELL). ELL pyramidal cells are organized in columns consisting of six neurons (one ON- and OFF-type superficial, intermediate, deep per column) that in turn project to the midbrain torus semicircularis (TS) and indirectly to higher brain areas mediating behavioral responses. Although all ELL pyramidal cells project to TS, only deep ON- and OFF-type cells also directly (gray) project to the nucleus praeeminentialis (nP). ELL pyramidal cells also receive large amounts of descending input (i.e., feedback; pink arrow) from nP.

We used an experimental preparation allowing for simultaneous multi-unit recordings from ELL pyramidal cells whereas the animal performed behavioral responses (Figure 1A). Specifically, behavior consisting of changes in EOD frequency that faithfully follow the stimulus’ detailed timecourse is recorded via a pair of electrodes located near the animal’s snout and tail (Figure 1A, top left). As mentioned above, such behavioral responses are thought to help individuals make themselves appear more dominant during social interactions.^31^ A high-density electrode array (Neuropixels probe) was inserted into the ELL region of the animal’s brain and allowed simultaneous multi-unit recordings from ELL pyramidal cells (Figure 1A, top right). Stimuli were delivered via a pair of electrodes located on either side of the animal (Figure 1A) and consisted of noisy AMs (Figure 1A, left gray) of the animal’s own EOD whose envelope (Figure 1A, left, green) varied sinusoidally at different frequencies within the behaviorally relevant range (0.001–1 Hz).^27^ Electrosensory afferents respond to EOD AMs and envelopes^63^^,^^64^ and make synaptic contact with ELL pyramidal cells that in turn project to the midbrain torus semicircularis (TS) and indirectly to higher brain areas whose activities mediate behavioral responses such as the nucleus electrosensorius as well as the prepacemaker nucleus (Figure 1B, black arrows). ELL pyramidal cells also receive large amounts of descending input in part from TS via nP either directly or indirectly via the eminentia granularis posterior (EGp; Figure 1B, pink arrows). Anatomical studies have shown that pyramidal cells are organized into columns within the ELL.^47^ Specifically, each column consists of six cells (ON and OFF-type superficial, intermediate, and deep; Figure 1B). Although only deep pyramidal cells directly project to nP, all six cell types project to TS^56^ (Figure 1B).

### Descending pathways mediate highly heterogeneous neural responses to envelope stimuli

**Figure 2:**
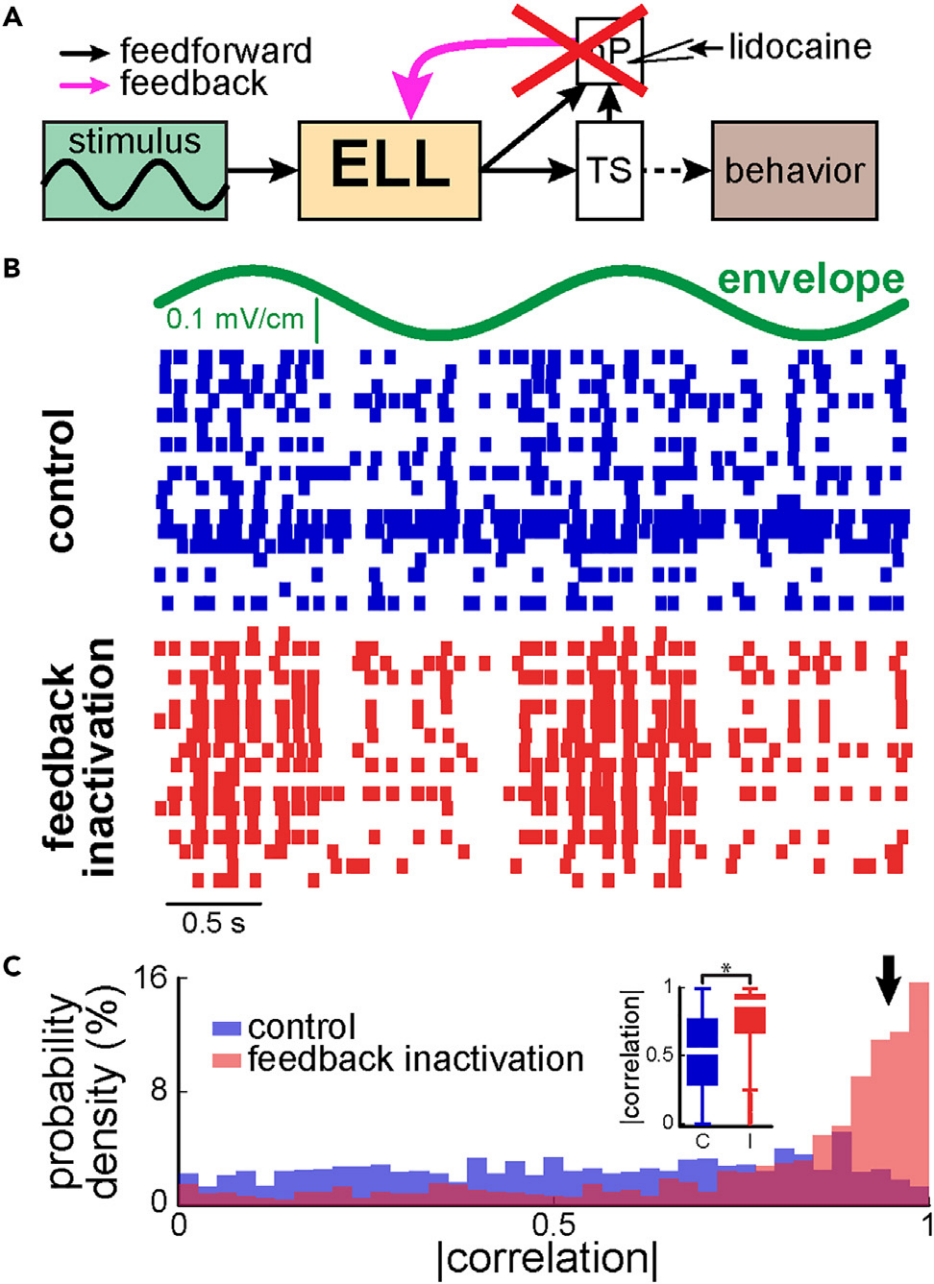
ELL pyramidal cells display highly heterogeneous responses to envelope stimuli that are strongly attenuated by pharmacological inactivation of feedback pathways (A) Simplified circuit diagram showing that pharmacological inactivation of feedback (pink arrow) onto ELL pyramidal cells was achieved by injecting lidocaine bilaterally into nP (red cross). (B) Envelope stimulus waveform (top, green) and raster plots showing the activities of the same ELL pyramidal cell population before (middle, blue) and after (bottom, red) feedback inactivation in response to this stimulus. Overall, it is seen that responses were much more similar to one another after feedback inactivation. (C) Histograms of the pairwise correlation coefficients between neural activities before (blue) and after (red) feedback inactivation. Note the increased probability of obtaining large correlation coefficient values near unity after feedback inactivation. Both distributions were significantly different from one another across our datasets (inset: two-sample Kolmogorov-Smirnov test, p = 1.04∗10^−6^, D = 39).

We first investigated how ELL pyramidal cell populations responded to sinusoidal envelopes with frequencies (0.1 Hz, 0.5 Hz, and 1 Hz) and contrasts (weak 9.1 ± 2.9%, intermediate 23.2 ± 2.9%, strong 49.7 ± 20.3%; see STAR Methods) that both varied within the natural range.^27^ Previous studies have shown that single ELL pyramidal cells display high-pass tuning to envelope stimuli, such that their sensitivities increase with envelope frequency.^29^^,^^41^ Descending pathways (i.e., feedback) were inactivated by injecting the sodium channel antagonist lidocaine into the nP bilaterally (see STAR Methods; Figure 2A), as done previously.^35^^,^^36^

We found that the spiking activities of ELL pyramidal cell populations in response to stimulation were highly heterogeneous as seen from a raster plot before feedback inactivation (i.e., “control”; Figure 2B, blue). Indeed, different neurons preferentially fired at different phases of the envelope cycle (Figure 2B, blue). We quantified response similarity by computing correlation coefficients between neural pairs (see STAR Methods). Overall, we found that correlation coefficient values were uniformly distributed under control conditions (Figure 2C, blue), which indicates a high degree of heterogeneity.

To investigate the nature of the mechanisms responsible for such heterogeneity, we first partitioned the recorded neurons into six groups (see STAR Methods). First, each cell was classified as either ON- or OFF-type based on responses to the carrier waveform (see STAR Methods). ON- and OFF-type cells can easily be distinguished from one another as they fire during opposite phases of the carrier waveform (Figures S1A and S1B). We note however that ON- and OFF-type strictly refers to the response profile to the carrier and *not* the envelope stimulus. Indeed, it is possible to find ON- and OFF-type cells that fire preferentially during opposite phases of the carrier yet respond similarly to the envelope (Figure S1A). Next, we partitioned ON- and OFF-type cells each into low, medium, and high firing rate based on their firing activity in the absence of stimulation that likely correspond to superficial, intermediate, and deep types (see STAR Methods). Overall, if the heterogeneity in envelope response observed for our dataset is primarily because of different cell types having different response properties, then we would expect that the firing activities of same type cells should be more similar (i.e., more highly correlated) than those of different type cells. Overall, we found similar largely uniform distribution for correlation coefficients between same type cells, which was comparable to that obtained for our entire dataset (compare Figures S2A–S2C, blue). Moreover, the distributions of correlation coefficients computed within cell type was not significantly different than that computed across cell types (Figure S2B). This indicates that response heterogeneity to envelope stimuli is not primarily because of differences across cell types. Rather, levels of response heterogeneity similar to that observed for the entire dataset is observed for each cell type.

Next, we investigated the effects of feedback inactivation. Overall, we found that ELL pyramidal cell activities in response to the same stimulus were much more similar after such inactivation (i.e., less heterogeneous; Figure 2B, compare blue and red raster plots showing population activity for the same neurons before and after feedback inactivation, respectively). Indeed, more correlation coefficient values near unity were observed after feedback inactivation (Figure 2C, red, arrow). As such, the distributions of correlation coefficients obtained before and after inactivation were significantly different from one another (two-sample Kolmogorov-Smirnov test; p = 1.04∗10^−6^, D = 39), indicating that such inactivation significantly reduces heterogeneity. Of interest, other attributes such as firing rate and sensitivity to the stimulus were not significantly altered by feedback inactivation (Figure S3). We note that the current study uses high-density arrays to record the activities of ELL pyramidal cell populations simultaneously in response to envelope stimuli, whereas previous studies instead relied on single unit recordings and focused on neurons that displayed clear responses to such stimuli. However, our results were similar to those obtained previously when only considering ELL pyramidal cells that displayed clear responses to envelope stimuli as quantified by neural sensitivity (see STAR Methods; compare Figures S4A and S4B of^35^).

How does feedback inactivation reduce heterogeneity? One possibility is that, because superficial cells tend to receive the largest amount of feedback and deep cells the least,^40^ feedback inactivation makes the envelope responses of superficial ELL pyramidal cells more similar to those of intermediate and deep cells. To test this prediction, we investigated the effects of feedback inactivation on all six cell types. Overall, only the activities of low firing rate ON-type cells became more similar to one another after feedback inactivation, whereas no significant change was observed for all five other cell types (Figure S5A). Moreover, the correlation coefficient distribution within cell type was significantly different than that across cell types (Figure S5B). As such, our results show that feedback inactivation does not reduce heterogeneity by making the responses of different cell types more similar to one another. Rather, such inactivation reduces heterogeneity selectively within one cell type (low firing rate ON-type) whereas the heterogeneity of all other cell types is not significantly affected.

### Neural heterogeneity serves a beneficial function by increasing decoding robustness

**Figure 3:**
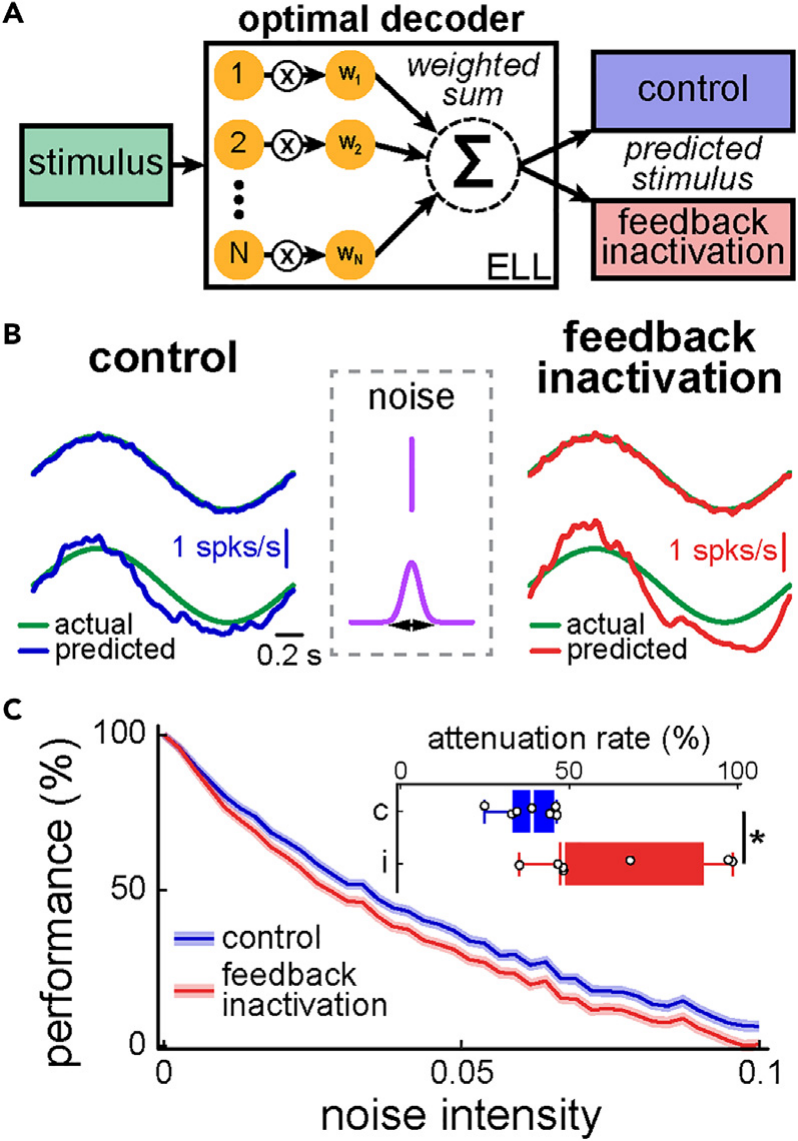
Heterogeneities are functionally beneficial, as decoders optimized to reconstruct the envelope stimulus’ detailed timecourse are more robust to noise addition before feedback inactivation (A) Schematic showing optimal decoding. Neural responses to the envelope are weighted and the weights are chosen such as to minimize the mean-squared error between the weighted sum of neural activities (i.e., the predicted stimulus) and the actual stimulus. (B) Actual (green) and predicted stimulus waveforms when the weights are optimized before (blue, top left) and after (red, top right) feedback inactivation. To test decoding robustness, independent normally distributed random numbers (i.e., noise) was added to each weight and the standard deviation of the distribution was progressively increased (see middle column). Increasing noise intensity increased the error between predicted and actual stimulus waveforms to a lesser extent before (blue, bottom left) than after (red, bottom right) feedback inactivation. (C) Performance as a function of noise intensity before (blue) and after (red) feedback inactivation. It is seen that performance is more greatly attenuated after feedback inactivation. Inset: The rate of increase of performance attenuation was greater after feedback inactivation (Wilcoxon signed rank test; p = 0.047, N = 7).

Does heterogeneity within ELL pyramidal cell activities in response to envelope stimuli serve a beneficial function (i.e., would such heterogeneity promote better information transmission about the stimulus’ detailed timecourse that is used by downstream brain areas to generate appropriate changes in EOD frequency)? To answer this question, we considered a decoder for which the envelope stimulus waveform is predicted from a weighted sum of neural activities (Figure 3A). We chose this decoding scheme because the animal’s behavioral responses consisting of changes in EOD frequency that faithfully follow the envelope stimulus, indicating that information as to the detailed timecourse must be retained along the brain pathways mediating this behavior.^31^ Because pyramidal cells are the sole output neurons of the ELL that respond to the stimuli used in the current study, their activities are most likely necessary to generate behavior. We included all six cell types in this analysis as they all project to higher brain areas.^56^ The weights were optimized such as to minimize the root-mean-square error (RMSE) between the predicted (i.e., the weighted sum of neural activities) and actual (i.e., the envelope) stimulus waveforms (see STAR Methods). To quantify the effect of heterogeneity, we compared the performance of this optimal decoder using experimental data from the same ELL pyramidal cell populations that were recorded before and after feedback inactivation. Overall, we found similar performance under both conditions (Figure 3B, compare top left and right traces). Thus, heterogeneity does not alter the performance of the optimal decoder. Of interest, in both cases, there was a significant negative correlation between weight magnitude and firing rate (Figures S6A and S6B), suggesting that low firing rate cells are assigned weights with larger magnitude than high firing rate cells. We thus compared weights for all six cell types and found that weight magnitude was highest for low firing rate ON-type cells (Figure S6C).

We then quantified the robustness of optimal decoders before and after feedback inactivation. To do so, we added noise to the optimal weights obtained in each case and progressively increased noise intensity (see STAR Methods). Overall, the decoding performance decreased in both cases but at a significantly higher rate after feedback inactivation (Figure 3C, compare blue and red, Figure 3C, inset; Wilcoxon signed rank test; p = 0.047, N = 7). Thus, our results show that heterogeneity serves a beneficial function, as the optimal decoder is more robust (i.e., the performance decreases less) to the addition of noise when compared to a more homogeneous population.

### Heterogeneity is functionally relevant to determine behavioral responses at the organismal level

**Figure 4:**
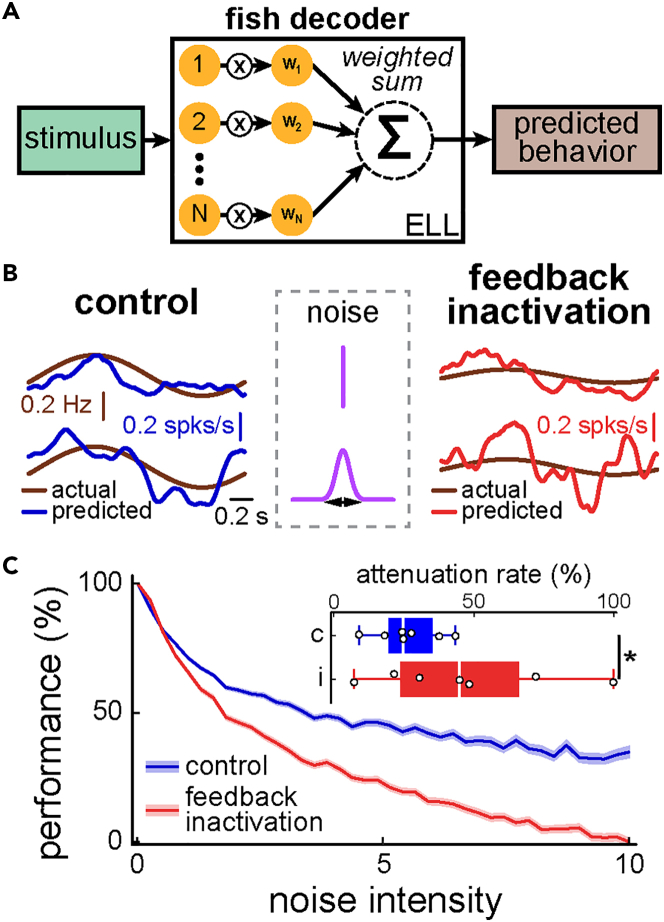
Downstream decoders take advantage of the beneficial function of heterogeneities to generate behavior (A) Schematic showing decoding. Neural responses to the envelope are weighted and the weights are chosen such as to minimize the mean-squared error between the weighted sum of neural activities (i.e., the predicted behavior) and the actual behavior. (B) Actual (brown) and predicted behavioral responses before (blue, top left) and after (red, top right) feedback inactivation. To test decoding robustness, independent normally distributed random numbers (i.e., noise) was added to each weight and the standard deviation of the distribution was progressively increased (see middle column). Increasing noise intensity increased the error between predicted and actual stimulus waveforms to a lesser extent before (blue, bottom left) than after (red, bottom right) feedback inactivation. (C) Performance as a function of noise intensity before (blue) and after (red) feedback inactivation. It is seen that performance is more greatly attenuated after feedback inactivation. Inset: The rate of increase of performance attenuation was greater after feedback inactivation. (Wilcoxon signed rank test; p = 0.016, N = 7).

Information carried by neural populations is only important to an organism if it is actively being decoded by neurons within downstream brain areas. Thus, although our results so far show that heterogeneity increases decoding robustness, they do not necessarily imply that the brain takes advantage of this benefit. This is because the decoder considered above, which is optimal for reconstructing the stimulus’ detailed timecourse, may or may not be representative of the decoding of ELL pyramidal cell activity that takes place within the animal’s brain to generate behavior. To address this important question, we considered whether decoding consisting of taking a weighted sum of neural activities can correctly predict the animal’s actual behavioral responses to envelope stimuli (Figures 4A and S7A). Overall, we found that the optimal decoder considered above, while correctly predicting behavioral responses before feedback inactivation (Figure S7B, compare light blue and brown traces), performed poorly at predicting the attenuated behavioral responses observed after feedback inactivation (Figure S7C, compare purple and brown traces). However, when considering instead a global decoder for which the weights are optimized taking all conditions from control data into account and are thus the same across control conditions (as opposed to being optimized for each condition independently), we found that such a global decoder much better predicted the changes in behavior that occur because of feedback inactivation (control: Figure S7B, compare dark blue and brown traces; after feedback inactivation: Figure S7C, compare red and brown traces). Moreover, such behavioral changes were similar to those observed previously (compare Figures S4B–S4E of^35^). As such, the overall performance across conditions was significantly better for the global than for the optimal decoder (Figure S7D). It should be furthermore noted that the weights obtained for the optimal decoder for a given condition generally performed poorly at predicting other conditions (Figure S8), which is indicative that weights associated with the optimal decoder before inactivation differed across stimulation conditions.

We took these results as indicative that the operation performed by the global decoder is representative of the decoding that takes place within the fish’s brain to generate behavior, and accordingly henceforth refer to this decoder as the “fish decoder”. The weights obtained from the fish decoder were significantly negatively correlated with firing rate (Figure S9A) and significantly positively correlated with those obtained from the fish decoder (Figure S9B). However, weight magnitude was not significantly different between all six cell types (Figure S9C).

We then compared the fish decoder’s performance at predicting behavioral responses before and after feedback inactivation. It is important to note that, unlike the optimal decoder considered previously, the weights are the same for both conditions as they are only derived from part of the control dataset (see STAR Methods). Overall, the fish decoder performed similarly at predicting behavioral responses before and after feedback inactivation (Figure 4B, compare top left and top right traces). We next quantified decoding robustness by adding noise in the same manner as that done above for the optimal decoder (Figure 4B, middle column, see STAR Methods). Overall, we found results that were qualitatively similar to those described above for the optimal decoder, in that decoding performance decreased at a comparatively larger rate after feedback inactivation (Figure 4C, compare blue and red curves, Figure 4C, inset; Wilcoxon signed rank test; p = 0.016, N = 7). Thus, we conclude that the decoding strategy used by the brain to generate behavior takes advantage of increased robustness when decoding from heterogeneous neural populations. Taken together, our results show that heterogeneity mediated by descending pathways serves a beneficial function by increasing decoding robustness, which is used by the brain to generate behavior. Below we discuss their implications.

## Discussion

### Summary of results

We investigated how response heterogeneity (i.e., differences in the spiking responses elicited by stimulation) influences information transmission by ELL pyramidal cell populations and how this information is decoded to generate behavior. We found that response heterogeneity was strongly attenuated by pharmacological inactivation of feedback pathways, but that other neural attributes such as stimulus sensitivity and firing rate were not affected. Of interest, ON and OFF-type ELL pyramidal cells with low, medium, and high firing rates, respectively, displayed similar heterogeneity levels before feedback inactivation, thereby providing strong evidence that heterogeneity was not primarily because of differences across cell types. Further analysis revealed that feedback inactivation primarily reduced heterogeneity within ON-type low firing rate cells, while having at best minimal effects on heterogeneity for the other types. By comparing the coding properties of the same ELL cell population before and after feedback inactivation, we showed that heterogeneity serves a beneficial function. Specifically, a decoder optimized to reconstruct the stimulus’ detailed timecourse was more robust to noise addition before feedback inactivation. Importantly, we show that downstream decoders within the fish brain that generate behavior are also more robust to noise addition before feedback inactivation. Taken together, our results provide experimental demonstration that heterogeneity serves a beneficial function that is used by the brain to generate behavior. Moreover, they provide evidence that response heterogeneity is actively promoted by neural circuits within sensory systems, which strongly suggests that such heterogeneity is not merely a by-product of noisy developmental processes.

### Role of feedback toward mediating response heterogeneities

Our results demonstrate that descending pathways promote response heterogeneity amongst the ELL pyramidal cell population by specifically increasing heterogeneity amongst ON-type low firing rate cells. It has previously been shown that ON-type cells correspond to the basilar pyramidal cells,^44^ whereas the prominent negative correlation between baseline firing rate and apical dendritic length^46^^,^^56^ strongly suggests that the low firing rate cell category considered here likely corresponds to the superficial type. If correct, then this implies that descending pathways actively enhance response heterogeneity to envelope stimuli primarily amongst superficial basilar pyramidal cells. All pyramidal cells including the superficial basilar type receive both direct and indirect sources of descending input.^54^^,^^65^ However, because of their prominent apical dendritic trees, superficial cells receive larger amounts of indirect descending input than their intermediate and deep counterparts.^47^ Indeed, previous studies have found that indirect descending input has a larger effect on the response properties of superficial pyramidal cells with respect to their intermediate and deep counterparts but used stimuli different than those used in the current study.^45^^,^^49^^,^^50^^,^^56^ Our results show response heterogeneity was not altered across cell types, indicating that ON-type low firing rate cell responses did not become more similar to that of other cell types overall including deep cells that are thought to receive little to no descending input. Previous studies have shown that both nP stellate cells projecting directly to ELL and nP multipolar cells projecting indirectly to ELL respond strongly to the envelope stimuli used in this study.^35^ Moreover, inactivation of direct descending input reduces whereas inactivation of indirect descending input instead tends to increase ELL pyramidal cell responses to envelope stimuli.^35^^,^^36^

As such, both direct and indirect feedback pathways could in theory contribute to increasing response heterogeneity amongst ON-type low firing rate cells. One possibility is that, because connections between nP stellate and ELL pyramidal cells are topographic,^54^ response heterogeneity to envelope stimuli in superficial basilar pyramidal cells is because of nP stellate cells displaying such heterogeneity in the first place. Another not necessarily mutually exclusive possibility is that superficial basilar ELL pyramidal cell response heterogeneities to envelope stimuli originate from differences in how they integrate feedback input from stellate cells. Supporting the latter possibility is the fact that ELL tuning to envelopes is mediated in part by small conductance calcium-activated potassium (SK) channels^34^ and that differences in expression of SK channels across ELL pyramidal cells can explain differences in their responses to envelopes.^41^^,^^53^ Although both basilar and non-basilar pyramidal cells express the SK1 channel subtype,^66^ only basilar pyramidal cells express the SK2 channel subtype.^66^^,^^67^ Because our results show that feedback inactivation reduced heterogeneity primarily for low firing rate ON-type cells, this suggests that envelope response heterogeneities might be because of differences in SK2 channel expression. SK2 channels tend to be located close to the soma,^66^ which is close to the proximal apical dendrites where direct descending input terminates.^47^ Yet another possibility is that indirect descending input causes increased envelope response heterogeneity amongst superficial basilar pyramidal cells. Previous studies have shown that this feedback is diffuse and thus is activated by stimuli impinging on a large fraction of the sensory epithelium,^47^^,^^54^^,^^57^ which is the case for the envelope stimuli used in this study. However, the fact that inactivation of indirect descending input tends to have overall similar effects on basilar and non-basilar pyramidal cells^49^^,^^50^^,^^56^ is at odds with our results although it should be noted that these previous studies used different stimuli.

Moreover, our results strongly suggest that feedback inactivation had at best minimal effects on envelope response heterogeneity amongst all other five ELL pyramidal cell types including OFF-type low firing rate cells. This suggests that response heterogeneity amongst these cell types is instead regulated by ascending processes. Such could include differences in ion channel expression such as aforementioned SK2 channels as well as differential input from local inhibitory interneurons. Overall, further studies are needed to understand the mechanisms by which descending pathways increase envelope response heterogeneity within superficial basilar ELL cells, as well as understanding the nature of the mechanisms by which ascending pathways regulate such heterogeneity for other ELL pyramidal cell types.

### Decoding information transmitted by ELL pyramidal cell populations

Our results show that a decoding operation consisting of taking a weighted sum of neural activities could accurately predict either the stimulus’ detailed timecourse or the animal’s behavioral responses. Moreover, they demonstrate that, in both cases, response heterogeneity was beneficial as the decoder was more robust to noise addition. However, there were important differences between the weights obtained when optimizing to reconstruct the stimulus’ detailed timecourse, or the animal’s behavioral responses independently across different stimulation conditions (i.e., the weights can change across conditions). Indeed, such “local” decoders performed poorly at predicting the changes in behavior that result from feedback inactivation, whereas a “global” decoder (i.e., the fish decoder) for which the weights are the same across conditions instead correctly predicted these. As such, it is likely that the decoding that is taking place within the fish brain is better represented by the fish decoder. This is because the changes in response properties and behavior that result from feedback inactivation occur on a timescale that is much smaller than those at which the system can adapt.^33^ A recent study has shown that a “global” decoder similar to that considered here is more representative of the strategies used by monkeys to discriminate between different stimuli,^68^ suggesting that this is a general strategy by which the brain decodes information transmitted by sensory neural populations.

Decoders consisting of taking weighted sums of neural activity are advantageous as they can convey much more information than the simple summation of neural activities.^15^^,^^69^^,^^70^^,^^71^^,^^72^^,^^73^^,^^74^ Such decoders have furthermore been used to either place a lower bound on the amount of information transmitted by neural populations or used to predict behavior.^68^^,^^75^^,^^76^^,^^77^ However, a major issue concerns how these can be implemented within the brain. First, because each neuron is assigned its own weight, these decoders must retain neural identity. Second, it is clear that the brain does not actually perform a weighted sum of neural activities to generate behavior, which implies that these are at best equivalent rather than actually equal to decoding by downstream brain areas. What operations are performed by the brain to decode ELL pyramidal cell activities to generate behavior? A potential solution to this problem has been proposed recently by having the weights proportional to response reliability.^78^ Our results suggest that the electrosensory system takes advantage of this solution, as we found that weight magnitude was negatively correlated with firing rate. As such, one possibility is that synaptic plasticity in downstream brain areas help regulate the weight assigned to each ELL pyramidal cell based on its firing activity. Indeed, previous studies have found that synapses from ELL neurons within the torus semicircularis display strong synaptic depression,^79^^,^^80^^,^^81^^,^^82^ which would attenuate synaptic strength more for neurons with higher firing activities. Our results showing that weights are more robust to noise addition when activities are heterogeneous provide further evidence that learning processes need not be fully accurate to reach an acceptable decoding performance. It is important to note that such a decoding scheme assumes that the firing activities of all six ELL pyramidal cell types are used to generate behavior. Such a scheme is physiologically realistic as all ELL pyramidal cells project to the downstream TS.^56^ The exact connectivity pattern between ELL and TS is not known although the fact that most TS neurons are ON-type,^83^ as opposed to there being equal numbers of ON- and OFF-type ELL pyramidal cells,^47^ suggests that it is asymmetric. Further studies are needed to verify these predictions and to understand how information carried by ELL pyramidal cell populations is decoded by downstream brain areas to generate behavior.

### Function of response heterogeneity

Our results demonstrate a novel function for descending pathways as they promote response heterogeneity within a specific ELL pyramidal cell type. As such, they provide evidence against the hypothesis that heterogeneity is merely the by-product of noisy developmental processes.^22^ For example, it is conceivable that such processes would cause heterogeneity in the level of expression of various membrane conductances that would in turn causes heterogeneity in responses to sensory input. Our results, however, show that this is not the case here, as response heterogeneities were strongly reduced after feedback inactivation. Moreover, the fact that such reduction was only seen for a specific cell type provides strong evidence that response heterogeneities amongst the ELL pyramidal cell populations are not “trivially” because of different cell types receiving differential amounts of descending input. This is because all six cell types displayed levels of heterogeneity similar not only to each other but also similar to that of the entire population. Rather, the fact that feedback inactivation specifically alters response heterogeneity amongst ON-type low firing rate cells suggests that this cell type serves a specific function. One intriguing possibility that sensory systems can regulate response heterogeneity to maximally take advantage of their beneficial function. Indeed, previous studies have shown that feedback pathways are highly plastic.^33^^,^^54^^,^^57^ As such, it is conceivable that an important function of descending pathways is to adjust response heterogeneity in ON-type low firing rate cells based on stimulus statistics and behavioral context to maximize their beneficial effects. Indeed, theoretical studies have shown that response heterogeneity is advantageous by giving rise to more robust learning.^21^^,^^22^ Moreover, a recent study has shown that ELL pyramidal cells can dynamically adapt their response properties such as to optimally encode stimuli with different statistics based on descending input.^33^ As such, one possibility is that descending pathways could regulate heterogeneity level depending on coding range, such that a higher level of heterogeneity would be observed for stimuli whose statistics require an increased coding range. Although theoretical studies predict that heterogeneity increases coding range,^84^ there is to our knowledge no experimental verification of this prediction to date. Further studies using stimuli with different statistics that require different coding ranges and investigating whether sensory adaptation gives rise to different levels of heterogeneity within a given sensory neural population are needed to test this prediction.

### Implication for other systems

It is likely that our results will be applicable to other systems. This is because, as mentioned above, response heterogeneity within a given cell type is observed ubiquitously across systems and species. Moreover, the envelope stimuli considered here are behaviorally relevant across sensory modalities (somatosensory:^85^; visual:^86^; vestibular:^87^^,^^88^; auditory:^89^). For example, envelopes found in natural auditory stimuli (e.g., speech) are particularly necessary for perception.^90^^,^^91^ In addition, descending pathways are found ubiquitously across systems and species and vastly outweigh feedforward input from the periphery.^92^^,^^93^^,^^94^^,^^95^ Although such descending input has important functional roles such as predictive coding^96^ or regulating how neural responsiveness to the stimulus,^97^^,^^98^ we provide here experimental evidence that feedback regulates response heterogeneity, which serves a beneficial function by increasing decoding robustness that is used by the electrosensory system. Our results are thus timely in that they show how descending pathways mediate sensory neural responses to and perception of behaviorally relevant stimulus features at the population level. Important commonalities between the electrosensory system and the visual, auditory, and vestibular systems of mammals (see^99^^,^^100^ for review) suggest that similar functions will be found in these systems as well.

### Limitations of the study

This study was carried out in the electrosensory system of weakly electric fish and, as such, the results may not be applicable to other systems and species.

## STAR★Methods

### Key resources table

**Table:** 

REAGENT or RESOURCE	SOURCE	IDENTIFIER
**Chemicals, peptides, and recombinant proteins**		

tubocurarine	Sigma-Aldrich	93750
lidocaine	AstraZeneca	N/A

**Deposited data**		

Datasets and analysis files	this paper	https://doi.org/10.6084/m9.figshare.19700185https://figshare.com/s/c0bc1a79cefa509278d3

**Experimental models: Organisms/strains**		

*Apteronotus leptorhynchus*	local suppliers	N/A

**Software**		

Spike2	Cambridge Electronic Designs	https://ced.co.uk
SpikeGLX	Janelia Research Campus	http://billkarsh.github.io/SpikeGLX
Phy2	CortexLab	https://github.com/cortex-lab/phy
MATLAB	Mathworks	https://www.mathworks.com

### Resource availability

#### Lead contact

Further information and requests for resources should be directed to the lead contact, Maurice J. Chacron (maurice.chacron@mcgill.ca).

#### Materials availability

This study did not generate new unique reagents.

### Experimental model and study participant details

All animal procedures were approved by McGill University’s animal care committee and were performed in accordance with the guidelines of the Canadian Council on Animal Care. Specimens of either sex of the wave-type weakly electric fish *Apteronotus leptorhynchus* (N = 7) were used exclusively in this study. Animals were purchased from tropical fish suppliers and were housed in groups (2–10 individuals) at controlled water temperatures (26–29°C) and conductivities (100–800 μS∗cm^−1^) according to published guidelines.^101^ It was not possible to determine the age of the specimens used.

### Method details

#### Surgery

Surgical procedures have been described in detail previously.^35^^,^^36^ Briefly, 0.1–0.5 mg of tubocurarine (Sigma) was injected intramuscularly to immobilize the animals for electrophysiology experiments. The animals were then transferred to an experimental tank (30 cm × 30 cm x 10 cm) containing water from the animal’s home tank and respired by a constant flow of oxygenated water through their mouth at a flow rate of 10 mL∗min^−1^. Subsequently, the animal’s head was locally anesthetized with lidocaine ointment (5%; AstraZeneca), the skull was partly exposed, and a small window (∼5 mm^2^) was opened over the hindbrain as well as bilaterally over both midbrains in order to access nucleus praeeminentialis (nP) for drug application as described below.^35^^,^^36^

#### Stimulation

The electric organ discharge of *Apteronotus leptorhynchus* is neurogenic, and therefore is not affected by injection of curare. All stimuli consisted of amplitude modulations (AMs) of the animal’s own EOD and were produced by triggering a function generator to emit one cycle of a sine wave for each zero crossing of the EOD as done previously.^45^ The frequency of the emitted sine wave was set slightly higher (∼30 Hz) than that of the EOD, which allowed the output of the function generator to be synchronized to the animal’s discharge. The emitted sine wave was subsequently multiplied with the desired AM waveform (MT3 multiplier; Tucker Davis Technologies), and the resulting signal was isolated from the ground (A395 linear stimulus isolator; World Precision Instruments). The isolated signal was then delivered through a pair of chloridized silver wire electrodes placed 15 cm away from the animal on either side of the recording tank perpendicular to the fish’s rostro-caudal axis at three different intensities (i.e., weak, intermediate, strong) and, as such, impinged upon most if not all of the sensory epithelium. As such, it is expected that these stimuli also impinged upon most if not the entire receptive field of each cell recorded from.^45^ This stimulation protocol has been used by multiple previous studies (see, e.g.,^52^^,^^58^). We used stimuli consisting of a 5–15 Hz noise (4^th^ order Butterworth) carrier waveform (i.e., AM) whose amplitude (i.e., envelope) was further modulated sinusoidally at 0.1 Hz, 0.5 Hz, and 1 Hz. This constitutes a behaviorally relevant range of frequencies which mimicked the envelope signals due to relative movement between two fish.^28^^,^^29^^,^^31^ We used a total of 9 different stimuli (3 envelope frequencies for each of weak, intermediate, and strong contrasts, respectively) with each stimulus lasting one envelope period (i.e., 10 s, 2 s, and 1 s for 0.1 Hz, 0.5 Hz, and 1 Hz, respectively). To measure contrast, a small dipole was placed close to the animal’s skin as done previously.^45^ Stimulus intensity was adjusted to produce changes in EOD amplitude that were <15% (i.e., weak contrast; 9.1 ± 2.9%), between 15% and 30% (i.e., intermediate contrast; 23.2 ± 2.9%), and >30% (i.e., strong contrast; 49.7 ± 20.3%) of the baseline level. It is important to realize that these contrasts are within the range that is routinely experienced by these fish under natural conditions.^30^ Each stimulus (i.e., the carrier waveform and its envelope) was presented 20 times (i.e., there are 20 trials in total each lasting one envelope period for each stimulus).

#### Recordings

Simultaneous extracellular recordings from ELL pyramidal cells were made using Neuropixels probes (Imec Inc.). We focused on pyramidal cells because these constitute the sole output neurons of the ELL.^42^ The probe was angled at approximately 15 deg with respect to vertical and advanced between 1500 and 2000 μm into the ELL with reference to the probe tip. Neurons were recorded at depths between 200 and 1400 μm from the brain surface and as such most likely included neurons within the lateral and centrolateral segments.^102^ Recordings were digitized at 30 kHz using spikeGLX (Janelia Research Campus) and stored on a hard drive for further analysis. Spikes were sorted using Kilosort^103^ and subsequently curated using the phy application (https://github.com/cortex-lab/phy).^104^^,^^105^ Overall, we recorded and sorted from a total of N = 134 ELL pyramidal cells across seven recording sessions. We note that not all neurons recorded from were stable over the entire stimulation protocol, such that data for a given set of stimuli was only available for a subpopulation (3 envelope frequencies at one contrast for session 1; 3 envelope frequencies at one contrast for session 2, 3 envelope frequencies for 3 contrasts for session 3, 3 envelope frequencies for 3 contrasts for session 4, 3 envelope frequencies for 2 contrasts for session 5, 3 envelope frequencies for 2 contrasts for session 6, and 3 envelope frequencies for 3 contrasts for session 7). The average baseline firing rate was 20.5 ± 11.1 spks/s (4.1–64.7 spks/s), which is similar to that reported in previous studies.^56^^,^^104^^,^^106^^,^^107^ We note that other cell types within the ELL (e.g., interneurons) typically display firing rates higher than those of ELL pyramidal cells.^108^

#### Pharmacological inactivation

To study the effects of descending pathways, we recorded ELL pyramidal cells and behavior for control in conjunction with their responses after bilateral lidocaine (1 mM) injection into nP. Drug application pipettes were made using single-barrel borosilicate capillary glass tubes (OD 1.5 mm, ID 0.86 mm, A-M Systems) and pulled by a vertical micropipette puller (Stoelting) to a fine tip that was subsequently broken to attain a tip diameter of approximately 5 μm. All pharmacological injections were performed approximately 1250–1750 μm below the surface where the nP is located using a duration of 130 ms at ∼20 psi using a Picospritzer (General Valve) as done previously.^35^^,^^36^^,^^59^ It is important to note that the effects of such inactivation start within at most 30 s after injection and last throughout the experiment. Since the induction timescale is much smaller than those associated with adaptation (e.g., sensory adaptation) in the electrosensory system,^33^ it is very unlikely that the electrosensory can adapt to this procedure.

#### Behavior

Previous studies have shown that the animal’s EOD frequency follows the detailed timecourse of the envelope stimulus.^31^^,^^33^ This behavioral response can easily be measured through a pair of electrodes located near the animal’s head and tail. Specifically, the time-varying EOD frequency was obtained from the zero crossings of the recorded EOD signal that were used to generate a binary sequence as described above that was then low pass filtered to obtain the time-varying EOD frequency as done previously.^33^

#### Data analysis

All data analysis was performed offline using custom written codes in Matlab software (MathWorks). Sorted and curated spike times for each neuron were converted into “binary” sequences sampled at 2 kHz. Specifically, the content of a given bin of width 0.5 ms was set to 1 if a spike occurred within it and 0 otherwise. We note that, as the binwidth (0.5 ms) used is smaller than the refractory period of ELL pyramidal cells,^47^^,^^109^ there can be at most one spike occurring during any given bin.

To quantify neural responses and relate them to the stimulus envelope, we used linear systems identifications techniques to compute the sensitivity and preferred phase to each envelope frequency as done previously.^34^^,^^35^ We first averaged over the cycles of the stimulus and fitted a sinewave to the resultant cycle histogram to determine the firing rate modulation. We then divided the amplitude of the firing rate modulation to the stimulus envelope amplitude observed in the dipole to obtain the gain to any given envelope frequency. Our filtered firing rates were obtained using a first-order Butterworth filter with cut-off frequencies of 0.15, 0.3, and 1.5 Hz for envelope frequencies 0.1, 0.5, and 1 Hz, respectively, as done in previous studies.^53^ The preferred phase was calculated from the sinusoidal fit to the phase histogram. Sensitivity and preferred phase values for behavior (i.e., the time-varying EOD frequency) were calculated using similar methods.^31^ We computed correlation coefficients between the filtered firing rates using the “corrcoef” function in Matlab, which were used to quantify similarity between them. The distributions of correlation coefficients obtained over all possible pairings were then obtained. Cells with sensitivity greater than 5 spks/s/(mV/cm) for the lowest envelope frequency (i.e., 0.1 Hz) under control conditions were deemed to clearly respond to the envelope stimulus.

For visualization purposes, we used a Kaiser filter as done previously^110^ to filter the binary sequence and the behavior to obtain estimates of the time dependent firing rate and time-varying EOD frequency, respectively. The cut-off frequency was set to be 20% higher than the stimulus frequency.

We classified each cell as being either ON-type or OFF-type based on the spike triggered-average (STA) to the 5-15 Hz carrier waveform as done previously.^107^^,^^111^^,^^112^ Specifically, ON- and OFF-type cells fire action potentials during carrier up- and downstrokes, respectively (see Figure S1). As such, the slope of the STA preceding the action potential will be positive for ON-type cells and negative for OFF-type cells. Analysis of our data shows that ON- and OFF-type cells have opposite phase preferences with respect to the carrier waveform (Figures S1A and S1B), which is expected based on previous studies.^45^ However, this is not the case when the envelope stimuli considered here are concerned. Indeed, it is possible to find both ON- and OFF-type cells that fire during carrier up- and downstrokes, respectively, but whose activities are both in phase with respect to the envelope (Figure S1A). As such, the labels ON- and OFF-type solely refer to responses to the carrier and not to the envelope.

Moreover, we used the fact that there is a strong correlation between dendritic morphology and baseline firing rate *in vivo*^46^^,^^56^ to distinguish between deep, intermediate, and superficial pyramidal cells. Specifically, we classified cells whose baseline firing rates were less than 15 spks/s as “low firing rate” (n_ON_: 29; n_OFF_: 21), cells whose baseline firing rates were greater than 25 spks/s as “high firing rate” (n_ON_: 15; n_OFF_: 20), and the rest as “medium firing rate” (n_ON_: 25; n_OFF_: 24). We note that these thresholds are similar to those used in previous studies.^47^^,^^48^^,^^49^^,^^50^^,^^106^^,^^113^ Because of the strong negative correlation between dendritic length and baseline firing rate, low firing rate cells will tend to have larger apical dendrites and thus likely correspond to the “superficial” class, while high firing rate cells will tend to have larger apical dendrites and thus likely correspond to the “deep” class. Finally, medium firing rate cells will tend to have apical dendrites whose length will be between that of the other two and thus likely correspond to the “intermediate” class.

#### Optimal decoder

We used a weighted sum of filtered firing rates to predict either the envelope stimulus or behavioral response from the activities of M neurons. Specifically, in each case, weights were chosen such as to minimize the root-mean-square error (RMSE) between the predicted and actual waveforms. For each condition (i.e., one envelope frequency delivered at a given contrast), an analytical formula for the weights can be obtained using the calculus of variations^114^:

(Equation 1) $$\vec{w}={Cov}^{-1}\left(f_{R}\right)\;\vec{\left\langle f_{R}\;S\right\rangle }$$

where $\vec{w}={\left(w_{1}\;\ldots \;w_{M}\right)}^{T}$ and $w_{i}$ is the weight of neuron *i*. Here ${Cov}^{-1}\left(f_{R}\right)$ is the inverse of the filtered firing rate covariance matrix, while $\vec{\left\langle f_{R}\;S\right\rangle }={\left(\left\langle f_{R1}S\right\rangle \;\ldots \;\left\langle f_{RN}S\right\rangle \right)}^{T}$ is the vector of cross-correlations between the trial-averaged filtered firing rate of neuron i, $f_{Ri}$, and S which is either the trial-averaged envelope stimulus waveform or the behavioral response (i.e., the time-varying EOD frequency). Note that the superscript “*T*” denotes transposition, while ⟨…⟩ denotes averaging over time. In practice, the mean of each trial-averaged filtered firing rate, as well as those of the trial-averaged envelope stimuli and behavioral responses were subtracted to compute weights and evaluate decoding performance. This decoder is considered “local” because the weights are optimized (and thus can be different) for each of the nine conditions (i.e., three different envelope frequencies at three different contrasts). Moreover, the weights were computed using the first 50% (i.e., the first ten trials) and performance was evaluated on the other 50% (i.e., the other ten trials) of the dataset. The performance of the decoder was estimated from the inverse of the RMSE. It is important to note that this optimal decoder can correctly predict behavioral responses when the weights are obtained for each condition. However, using the weights obtained for a given condition (e.g., 0.5 Hz envelope frequency at intermediate contrast) to predict behavioral responses for a different stimulation condition gave poor results overall (Figure S8).

#### Fish decoder

Because the optimal decoder described above could not successfully predict changes in behavioral responses following feedback inactivation (Figure S7), we instead considered a “global” decoder for which the weights are the same for all nine conditions to predict behavior. In this case, the weights were chosen such as to minimize the sum of the RMSEs obtained for each condition. Because it is no longer possible to obtain an analytical formula for the weights in this case, we used a differential evolution algorithm that is described below in order to find the weights numerically.^105^^,^^115^ The weights were otherwise obtained similarly to what is described above. In particular, the evolution algorithm was trained using the first 50% of trials using control (i.e., before feedback inactivation) only. The performance of the decoder was then tested on the other 50% of trials before feedback inactivation and all trials obtained after feedback inactivation. We found that this “global” decoder could correctly predict the changes in behavior resulting from feedback inactivation (Figure S7), indicating that this decoding operation is representative of what occurs in the fish brain. We thus refer to this decoder as the “fish decoder” in the text.

#### Differential evolution algorithm

The evolution algorithm consists of having the weights “evolve” over a series of iterations (i.e., “generations”) in order to minimize the sum of RMSEs for all nine conditions. In keeping with the notation used in previous studies,^105^^,^^115^ we denote $X_{k}^{r}$ as the weight of neuron *r* for generation *k*. First, the population of weights is randomly initialized with values that are uniformly distributed with zero mean. For each neuron at every generation, a new weight is constructed by two operations consisting of "differentiation" and "recombination". In differentiation, the new weight of neuron r vector $X_{k,trial}^{r}$ is built by combining the weights of three other neurons $X_{k}^{r_{1}}$, $X_{k}^{r_{2}}$, and $X_{k}^{r_{3}}$, where r_1_ ≠ r_2_ ≠ r_3_:

(Equation 2) $$X_{k,trial}^{r}=X_{k}^{r_{1}}+\left(X_{k}^{r_{2}}-X_{k}^{r_{3}}\right)F\forall r=1,\ldots ,M$$

where the differential weight *F* = 0.5, and the three other neurons are chosen based on a probability distribution that is preferentially weighted for more fit (i.e., lower fitness score) individuals:

(Equation 3) $$p_{k}^{r}=\lambda \;\exp\left(\frac{{-F}_{fit}\left(X_{k}^{r}\right)}{\max_{\forall j}\left({1-F}_{fit}\left(X_{k}^{j}\right)\right)}\right)\forall r=1,\ldots ,M$$

where *λ* is a normalization constant such that the sum of probability values is equal to one. Recombination is then performed as follows:

(Equation 4) $$X_{mut}^{r}=\left\{ \begin{array}{c} X_{k,trial}^{r},if\;u<CR\\ X_{k}^{r},otherwise \end{array}\right.\forall r=1,\ldots ,N$$

where *u* is a random variable generated from a uniform distribution *U*(0,1) and with crossover probability *CR* = 0.9. Selection is finally performed to produce the next generation via:

(Equation 5) $$X_{k+1}^{r}=\left\{ \begin{array}{c} X_{mut}^{r},if\;F_{fit}\left(X_{mut}^{r}\right)<F_{fit}\left(X_{k}^{r}\right)\\ X_{k}^{r},otherwise \end{array}\right.\forall r=1,\ldots ,N$$

In this study, the fitness function was defined as:

(Equation 6) $$F_{fit}\left(X_{k}^{r}\right)=\sum_{i=1}^{9}{RMSE}_{i}$$

where ${RMSE}_{i}$ is the mean square error based on the actual behavioral responses and those predicted from the weights $X_{k}^{r}$ for condition *i*. The evolution algorithm was terminated if the change in population discrimination performance for the previous 20 iterations was on average below a threshold value of 0.001, and the weights were taken as those obtained for the last generation.

#### Noise and decoding performance

To quantify the robustness of the decoders described above, we added noise to the weights and quantified the change in decoding performance. Specifically, independent and normally distributed random variables with zero mean and standard deviation σ (i.e., the noise intensity) were added to the weights (i.e., the optimal weights) that minimized either the RMSE (in the case of the optimal decoder) and the sum of RMSEs over all conditions (in the case of the fish decoder). The RMSEs or the sum of RMSEs were then recomputed using the weights with noise added, and the effect on performance was quantified using:

(Equation 7) 100×(1RMSE)σ(1RMSE)σ=0

where “RMSE” is either the individual RMSE or the sum of RMSEs. The numerator corresponds to the performance obtained with the addition of noise with noise intensity σ, while the denominator corresponds to the performance obtained with no noise added (i.e., σ=0). In practice, performance with noise added was averaged over 30 realizations of the noise for each intensity. Because the weights obtained after noise addition are no longer optimized, the performance of the resulting decoder will be less than that obtained when no noise is added, implying that the ratio in Equation 7 is always less than one. It is also expected that, as noise intensity increases, RMSE will increase and thus performance will decrease. Thus, we computed the rate at which performance decreases by fitting a straight line to log-transformed performance as a function of noise intensity obtained before and after feedback inactivation in the case of the fish decoder. In the case of the optimal decoder, the rate at which performance decreases was obtained by fitting a straight line to the performance as a function of the log-transformed noise intensity obtained before and after feedback inactivation. Note that, for either of the fish or optimal decoders, the data obtained before and after feedback inactivation were processed in the same manner. The difference in the rate of decrease obtained before and after feedback inactivation was computed for each dataset, and statistical significance was assessed using a Wilcoxon signed rank test.

### Quantification and statistical analysis

Values are reported as mean ± SEM throughout. Differences between distributions were assessed using Kolmogorov-Smirnov tests unless otherwise stated. In general, since data was obtained from the same ELL pyramidal cells before and after inactivation, a paired t-test was used when data was normally distributed as assessed from a Lilliefors test using the function “lillietest” in MATLAB. Otherwise, a Wilcoxon signed rank test was performed on the differences (i.e., that between values obtained before and after feedback inactivation). Pearson’s correlation coefficient values were computed using the function “corrcoef” in MATLAB.

## Data Availability

• Spike train data have been deposited at figshare and are publicly available as of the date of publication. DOIs are listed in the key resources table. • All original code has been deposited at figshare and is publicly available as of the date of publication. DOIs are listed in the key resources table. • Any additional information required to reanalyze the data reported in this paper is available from the lead contact upon request.

## References

[1] Averbeck B.B., Latham P.E., Pouget A. (2006). Neural correlations, population coding and computation. Nat. Rev. Neurosci..

[2] Kohn A., Coen-Cagli R., Kanitscheider I., Pouget A. (2016). Correlations and Neuronal Population Information. Annu. Rev. Neurosci..

[3] Urai A.E., Doiron B., Leifer A.M., Churchland A.K. (2022). Large-scale neural recordings call for new insights to link brain and behavior. Nat. Neurosci..

[4] Panzeri S., Moroni M., Safaai H., Harvey C.D. (2022). The structures and functions of correlations in neural population codes. Nat. Rev. Neurosci..

[5] Hubel D.H., Wiesel T.N. (1962). Receptive fields, binocular interaction and functional architecture in the cat's visual cortex. J. Physiol..

[6] Ringach D.L., Shapley R.M., Hawken M.J. (2002). Orientation selectivity in macaque V1: diversity and laminar dependence. J. Neurosci..

[7] Kilgard M.P., Merzenich M.M. (1999). Distributed representation of spectral and temporal information in rat primary auditory cortex. Hear. Res..

[8] Staiger J.F., Flagmeyer I., Schubert D., Zilles K., Kötter R., Luhmann H.J. (2004). Functional diversity of layer IV spiny neurons in rat somatosensory cortex: quantitative morphology of electrophysiologically characterized and biocytin labeled cells. Cereb. Cortex.

[9] Lee D., Port N.L., Kruse W., Georgopoulos A.P. (1998). Variability and correlated noise in the discharge of neurons in motor and parietal areas of the primate cortex. J. Neurosci..

[10] Chelaru M.I., Dragoi V. (2008). Efficient coding in heterogeneous neuronal populations. Proc. Natl. Acad. Sci. USA.

[11] Mejias J.F., Longtin A. (2012). Optimal heterogeneity for coding in spiking neural networks. Phys. Rev. Lett..

[12] Shamir M., Sompolinsky H. (2006). Implications of neuronal diversity on population coding. Neural Comput..

[13] Hunsberger E., Scott M., Eliasmith C. (2014). The competing benefits of noise and heterogeneity in neural coding. Neural Comput..

[14] Zeldenrust F., Gutkin B., Denéve S. (2021). Efficient and robust coding in heterogeneous recurrent networks. PLoS Comput. Biol..

[15] Osborne L.C., Palmer S.E., Lisberger S.G., Bialek W. (2008). The neural basis for combinatorial coding in a cortical population response. J. Neurosci..

[16] Padmanabhan K., Urban N.N. (2010). Intrinsic biophysical diversity decorrelates neuronal firing while increasing information content. Nat. Neurosci..

[17] Ecker A.S., Berens P., Tolias A.S., Bethge M. (2011). The effect of noise correlations in populations of diversely tuned neurons. J. Neurosci..

[18] Tripathy S.J., Padmanabhan K., Gerkin R.C., Urban N.N. (2013). Intermediate intrinsic diversity enhances neural population coding. Proc. Natl. Acad. Sci. USA.

[19] Lengler J., Jug F., Steger A. (2013). Reliable neuronal systems: the importance of heterogeneity. PLoS One.

[20] Sachdeva P.S., Livezey J.A., DeWeese M.R. (2020). Heterogeneous Synaptic Weighting Improves Neural Coding in the Presence of Common Noise. Neural Comput..

[21] Kilpatrick Z.P., Ermentrout B., Doiron B. (2013). Optimizing working memory with heterogeneity of recurrent cortical excitation. J. Neurosci..

[22] Perez-Nieves N., Leung V.C.H., Dragotti P.L., Goodman D.F.M. (2021). Neural heterogeneity promotes robust learning. Nat. Commun..

[23] Bell C., Maler L., Bullock T.H., Hopkins C.D., Popper A.N., Fay R.R. (2005). Electroreception.

[24] Heiligenberg W. (1991).

[25] Turner R.W., Maler L., Burrows M. (1999). Electroreception and electrocommunication. J. Exp. Biol..

[26] Yu N., Hupé G., Garfinkle C., Lewis J.E., Longtin A. (2012). Coding conspecific identity and motion in the electric sense. PLoS Comput. Biol..

[27] Fotowat H., Harrison R.R., Krahe R. (2013). Statistics of the electrosensory input in the freely swimming weakly electric fish Apteronotus leptorhynchus. J. Neurosci..

[28] Stamper S.A., Fortune E.S., Chacron M.J. (2013). Perception and coding of envelopes in weakly electric fishes. J. Exp. Biol..

[29] Metzen M.G., Chacron M.J., Carlson B., Sisneros J., Popper A., Fay R. (2019). Electroreception: Fundamental Insights from Comparative Approaches.

[30] Yu N., Hupe G., Longtin A., Lewis J.E. (2019). Electrosensory Contrast Signals for Interacting Weakly Electric Fish. Front. Integr. Neurosci..

[31] Metzen M.G., Chacron M.J. (2014). Weakly electric fish display behavioral responses to envelopes naturally occurring during movement: implications for neural processing. J. Exp. Biol..

[32] Fortune E.S., Andanar N., Madhav M., Jayakumar R.P., Cowan N.J., Bichuette M.E., Soares D. (2020). Spooky Interaction at a Distance in Cave and Surface Dwelling Electric Fishes. Front. Integr. Neurosci..

[33] Huang C.G., Metzen M.G., Chacron M.J. (2019). Descending pathways mediate adaptive optimized coding of natural stimuli in weakly electric fish. Sci. Adv..

[34] Huang C.G., Zhang Z.D., Chacron M.J. (2016). Temporal decorrelation by SK channels enables efficient neural coding and perception of natural stimuli. Nat. Commun..

[35] Huang C.G., Metzen M.G., Chacron M.J. (2018). Feedback optimizes neural coding and perception of natural stimuli. Elife.

[36] Metzen M.G., Huang C.G., Chacron M.J. (2018). Descending pathways generate perception of and neural responses to weak sensory input. PLoS Biol..

[37] Marquez M.M., Chacron M.J. (2020). Serotonin modulates optimized coding of natural stimuli through increased neural and behavioural responses via enhanced burst firing. J. Physiol..

[38] Marquez M.M., Chacron M.J. (2020). Serotonergic Modulation of Sensory Neuron Activity and Behavior in Apteronotus albifrons. Front. Integr. Neurosci..

[39] Marsat G., Longtin A., Maler L. (2012). Cellular and circuit properties supporting different sensory coding strategies in electric fish and other systems. Curr. Opin. Neurobiol..

[40] Krahe R., Maler L. (2014). Neural maps in the electrosensory system of weakly electric fish. Curr. Opin. Neurobiol..

[41] Huang C.G., Chacron M.J. (2017). SK channel subtypes enable parallel optimized coding of behaviorally relevant stimulus attributes: A review. Channels.

[42] Maler L. (1979). The posterior lateral line lobe of certain gymnotiform fish. Quantitative light microscopy. J. Comp. Neurol..

[43] Maler L., Sas E.K., Rogers J. (1981). The cytology of the posterior lateral line lobe of high frequency weakly electric fish (Gymnotidae): Differentiation and synaptic specificity in a simple cortex. J. Comp. Neurol..

[44] Saunders J., Bastian J. (1984). The physiology and morphology of two classes of electrosensory neurons in the weakly electric fish *Apteronotus Leptorhynchus*. J. Comp. Physiol..

[45] Bastian J., Chacron M.J., Maler L. (2002). Receptive field organization determines pyramidal cell stimulus-encoding capability and spatial stimulus selectivity. J. Neurosci..

[46] Bastian J., Nguyenkim J. (2001). Dendritic modulation of burst-like firing in sensory neurons. J. Neurophysiol..

[47] Maler L. (2009). Receptive field organization across multiple electrosensory maps. I. Columnar organization and estimation of receptive field size. J. Comp. Neurol..

[48] Avila-Akerberg O., Krahe R., Chacron M.J. (2010). Neural heterogeneities and stimulus properties affect burst coding in vivo. Neuroscience.

[49] Chacron M.J. (2006). Nonlinear information processing in a model sensory system. J. Neurophysiol..

[50] Chacron M.J., Maler L., Bastian J. (2005). Feedback and feedforward control of frequency tuning to naturalistic stimuli. J. Neurosci..

[51] Marsat G., Maler L. (2010). Neural heterogeneity and efficient population codes for communication signals. J. Neurophysiol..

[52] Marsat G., Proville R.D., Maler L. (2009). Transient signals trigger synchronous bursts in an identified population of neurons. J. Neurophysiol..

[53] Huang C.G., Chacron M.J. (2016). Optimized Parallel Coding of Second-Order Stimulus Features by Heterogeneous Neural Populations. J. Neurosci..

[54] Berman N.J., Maler L. (1999). Neural architecture of the electrosensory lateral line lobe: adaptations for coincidence detection, a sensory searchlight and frequency-dependent adaptive filtering. J. Exp. Biol..

[55] Bastian J. (1986). Gain control in the electrosensory system mediated by descending inputs to the electrosensory lateral line lobe. J. Neurosci..

[56] Bastian J., Chacron M.J., Maler L. (2004). Plastic and non-plastic cells perform unique roles in a network capable of adaptive redundancy reduction. Neuron.

[57] Bastian J. (1999). Plasticity of feedback inputs in the apteronotid electrosensory system. J. Exp. Biol..

[58] Mejias J.F., Marsat G., Bol K., Maler L., Longtin A. (2013). Learning contrast-invariant cancellation of redundant signals in neural systems. PLoS Comput. Biol..

[59] Clarke S.E., Maler L. (2017). Feedback Synthesizes Neural Codes for Motion. Curr. Biol..

[60] Kim C., Chacron M.J. (2020). Lower Baseline Variability Gives Rise to Lower Detection Thresholds in Midbrain than Hindbrain Electrosensory Neurons. Neuroscience.

[61] Middleton J.W., Longtin A., Benda J., Maler L. (2006). The cellular basis for parallel neural transmission of a high-frequency stimulus and its low-frequency envelope. Proc. Natl. Acad. Sci. USA.

[62] Hofmann V., Chacron M.J. (2019). Novel Functions of Feedback in Electrosensory Processing. Front. Integr. Neurosci..

[63] Metzen M.G., Chacron M.J. (2015). Neural heterogeneities determine response characteristics to second-but not first-order stimulus features. J. Neurosci..

[64] Bastian J. (1981). Electrolocation I. How the electroreceptors of *Apteronotus albifrons* code for moving objects and other electrical stimuli. J. Comp. Physiol..

[65] Sas E., Maler L. (1983). The nucleus praeeminentialis: A golgi study of a feedback center in the electrosensory system of gymnotid fish. J. Comp. Neurol..

[66] Ellis L.D., Maler L., Dunn R.J. (2008). Differential distribution of SK channel subtypes in the brain of the weakly electric fish Apteronotus leptorhynchus. J. Comp. Neurol..

[67] Ellis L.D., Mehaffey W.H., Harvey-Girard E., Turner R.W., Maler L., Dunn R.J. (2007). SK channels provide a novel mechanism for the control of frequency tuning in electrosensory neurons. J. Neurosci..

[68] Ni A.M., Huang C., Doiron B., Cohen M.R. (2022). A general decoding strategy explains the relationship between behavior and correlated variability. Elife.

[69] Reich D.S., Mechler F., Victor J.D. (2001). Independent and redundant information in nearby cortical neurons. Science.

[70] Seung H.S., Sompolinsky H. (1993). Simple models for reading neuronal population codes. Proc. Natl. Acad. Sci. USA.

[71] Sanger T.D. (1996). Probability density estimation for the interpretation of neural population codes. J. Neurophysiol..

[72] Abbott L.F., Dayan P. (1999). The Effect of Correlated Variability on the Accuracy of a Population Code. Neural Comput..

[73] Liu S., Gu Y., DeAngelis G.C., Angelaki D.E. (2013). Choice-related activity and correlated noise in subcortical vestibular neurons. Nat. Neurosci..

[74] Pitkow X., Liu S., Angelaki D.E., DeAngelis G.C., Pouget A. (2015). How Can Single Sensory Neurons Predict Behavior?. Neuron.

[75] Hohl S.S., Chaisanguanthum K.S., Lisberger S.G. (2013). Sensory population decoding for visually guided movements. Neuron.

[76] Yates J.L., Katz L.N., Levi A.J., Pillow J.W., Huk A.C. (2020). A simple linear readout of MT supports motion direction-discrimination performance. J. Neurophysiol..

[77] Zavitz E., Price N.S.C. (2019). Weighting neurons by selectivity produces near-optimal population codes. J. Neurophysiol..

[78] Sharpee T.O., Berkowitz J.A. (2019). Linking neural responses to behavior with information-preserving population vectors. Curr. Opin. Behav. Sci..

[79] Chacron M.J., Toporikova N., Fortune E.S. (2009). Differences in the time course of short-term depression across receptive fields are correlated with directional selectivity in electrosensory neurons. J. Neurophysiol..

[80] Fortune E.S., Rose G.J. (1997). Passive and active membrane properties contribute to the temporal filtering properties of midbrain neurons *in vivo*. J. Neurosci..

[81] Fortune E.S., Rose G.J. (2000). Short-term synaptic plasticity contributes to the temporal filtering of electrosensory information. J. Neurosci..

[82] Fortune E.S., Rose G.J. (2001). Short-term synaptic plasticity as a temporal filter. Trends Neurosci..

[83] McGillivray P., Vonderschen K., Fortune E.S., Chacron M.J. (2012). Parallel coding of first- and second-order stimulus attributes by midbrain electrosensory neurons. J. Neurosci..

[84] Lánský P., Getz W.M. (2001). Receptor heterogeneity and its effect on sensitivity and coding range in olfactory sensory neurons. Bull. Math. Biol..

[85] Lundstrom B.N., Fairhall A.L., Maravall M. (2010). Multiple timescale encoding of slowly varying whisker stimulus envelope in cortical and thalamic neurons in vivo. J. Neurosci..

[86] Baker C.L., Mareschal I. (2001). Processing of second-order stimuli in the visual cortex. Prog. Brain Res..

[87] Metzen M.G., Jamali M., Carriot J., Ávila-Ǻkerberg O., Cullen K.E., Chacron M.J. (2015). Coding of envelopes by correlated but not single-neuron activity requires neural variability. Proc. Natl. Acad. Sci. USA.

[88] Carriot J., Jamali M., Cullen K.E., Chacron M.J. (2017). Envelope statistics of self-motion signals experienced by human subjects during everyday activities: Implications for vestibular processing. PLoS One.

[89] Joris P.X., Schreiner C.E., Rees A. (2004). Neural processing of amplitude-modulated sounds. Physiol. Rev..

[90] Shannon R.V., Zeng F.G., Kamath V., Wygonski J., Ekelid M. (1995). Speech recognition with primarily temporal cues. Science.

[91] Shannon R.V., Zeng F.G., Wygonski J. (1998). Speech recognition with altered spectral distribution of envelope cues. J. Acoust. Soc. Am..

[92] Cajal R.S. (1909).

[93] Holländer H. (1970). The projection from the visual cortex to the lateral geniculate body (LGB). An experimental study with silver impregnation methods in the cat. Exp. Brain Res..

[94] Ostapoff E.M., Morest D.K., Potashner S.J. (1990). Uptake and retrograde transport of [^3^H]GABA from the cochlear nucleus to the superior olive in the guinea pig. J. Chem. Neuroanat..

[95] Sherman S.M., Guillery R.W. (2002). The role of the thalamus in the flow of information to the cortex. Philos. Trans. R. Soc. Lond. B Biol. Sci..

[96] Bastos A.M., Usrey W.M., Adams R.A., Mangun G.R., Fries P., Friston K.J. (2012). Canonical microcircuits for predictive coding. Neuron.

[97] Hupé J.M., James A.C., Payne B.R., Lomber S.G., Girard P., Bullier J. (1998). Cortical feedback improves discrimination between figure and background by V1, V2 and V3 neurons. Nature.

[98] Chance F.S., Abbott L.F., Reyes A.D. (2002). Gain Modulation from Background Synaptic Input. Neuron.

[99] Clarke S.E., Longtin A., Maler L. (2015). Contrast coding in the electrosensory system: parallels with visual computation. Nat. Rev. Neurosci..

[100] Bullock T.H., Hopkins C.D., Popper A.N., Fay R.R. (2005).

[101] Hitschfeld E.M., Stamper S.A., Vonderschen K., Fortune E.S., Chacron M.J. (2009). Effects of restraint and immobilization on electrosensory behaviors of weakly electric fish. Lab. Anim. Res..

[102] Maler L., Sas E., Johnston S., Ellis W. (1991). An atlas of the brain of the weakly electric fish *Apteronotus Leptorhynchus*. J. Chem. Neuroanat..

[103] Steinmetz N.A., Aydin C., Lebedeva A., Okun M., Pachitariu M., Bauza M., Beau M., Bhagat J., Böhm C., Broux M. (2021). Neuropixels 2.0: A miniaturized high-density probe for stable, long-term brain recordings. Science.

[104] Wang Z., Chacron M.J. (2021). Synergistic population coding of natural communication stimuli by hindbrain electrosensory neurons. Sci. Rep..

[105] Metzen M.G., Chacron M.J. (2021). Population coding of natural electrosensory stimuli by midbrain neurons. J. Neurosci..

[106] Bastian J., Courtright J. (1991). Morphological correlates of pyramidal cell adaptation rate in the electrosensory lateral line lobe of weakly electric fish. J. Comp. Physiol..

[107] Haggard M., Chacron M.J. (2023). Coding of object location by heterogeneous neural populations with spatially dependent correlations in weakly electric fish. PLoS Comp Biol.

[108] Bastian J., Courtright J., Crawford J. (1993). Commissural neurons of the electrosensory lateral line lobe of *Apteronotus Leptorhynchus*. Morphological and physiological characteristics. J. Comp. Physiol..

[109] Toporikova N., Chacron M.J. (2009). Dendritic SK channels gate information processing *in vivo* by regulating an intrinsic bursting mechanism seen *in vitro*. J. Neurophysiol..

[110] Cherif S., Cullen K.E., Galiana H.L. (2008). An improved method for the estimation of firing rate dynamics using an optimal digital filter. J. Neurosci. Methods.

[111] Martinez D., Metzen M.G., Chacron M.J. (2016). Electrosensory processing in Apteronotus albifrons: implications for general and specific neural coding strategies across wave-type weakly electric fish species. J. Neurophysiol..

[112] Hofmann V., Chacron M.J. (2020). Neural On- and Off-type heterogeneities improve population coding of envelope signals in the presence of stimulus-induced noise. Sci. Rep..

[113] Sproule M.K.J., Chacron M.J. (2017). Electrosensory neural responses to natural electro-communication stimuli are distributed along a continuum. PLoS One.

[114] Rieke F., Warland D., de Ruyter van Steveninck R.R., Bialek W. (1996).

[115] Aumentado-Armstrong T., Metzen M.G., Sproule M.K.J., Chacron M.J. (2015). Electrosensory Midbrain Neurons Display Feature Invariant Responses to Natural Communication Stimuli. PLoS Comput. Biol..

