# Supplementary material for: Descending pathways increase sensory neural response heterogeneity to facilitate decoding and behavior
Source: iScience. 2023 Jun 15;26(7):107139. doi: 10.1016/j.isci.2023.107139 (PMC10320509; doi:10.1016/j.isci.2023.107139)
Supplement: Document S2. Figures S1–S9 [file mmc1.pdf]

iScience, Volume *26*

Supplemental information

Descending pathways increase sensory neural response heterogeneity to facilitate decoding and behavior

Michael G. Metzen and Maurice J. Chacron

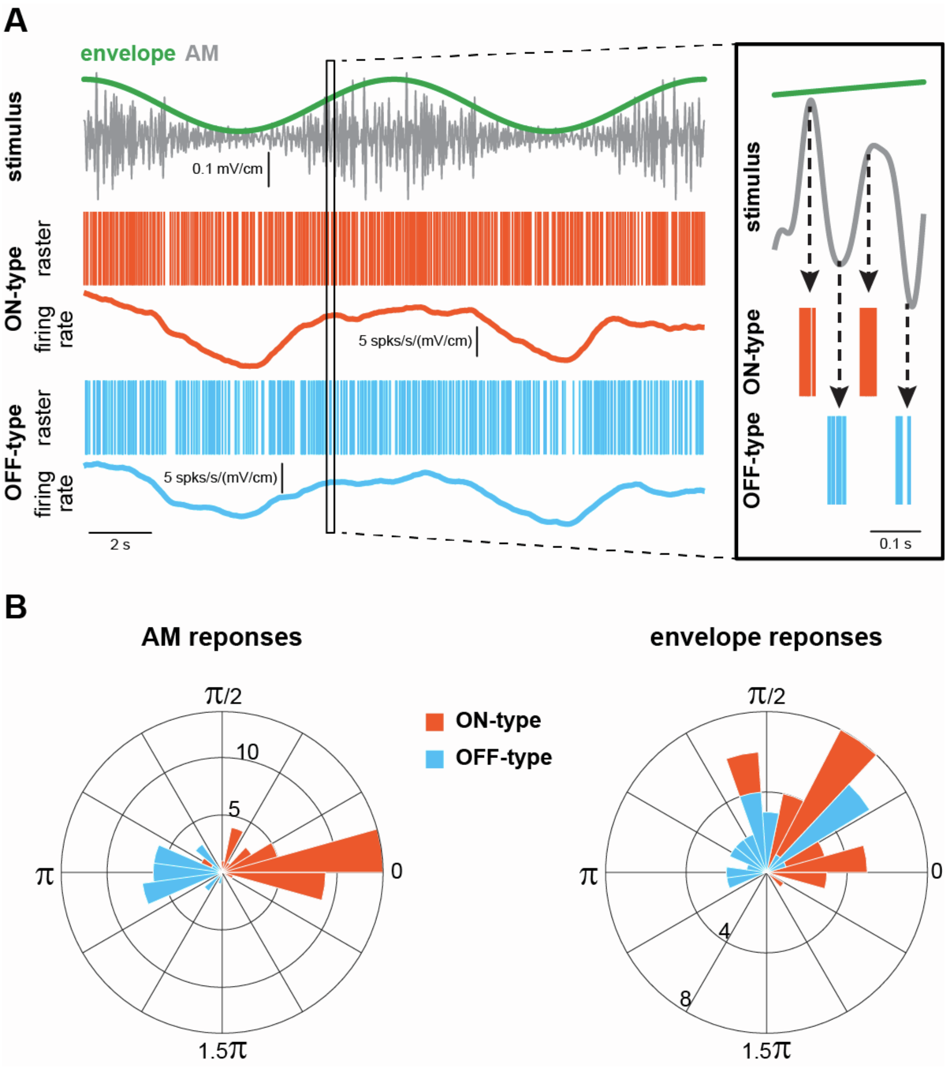

Supplementary Figure 1 related to Figure 1: *Responses of ON- and OFF-type ELL pyramidal cells to the carrier and envelope waveforms.* **(A)** *Top:* Stimulus consisting of the amplitude modulation waveform (i.e., carrier, gray) and the envelope (green). *Middle:* Spike train and time-dependent firing rate for an example ON-type cell. *Bottom*: Same as middle, but for an example OFF-type cell. Both ON- and OFF-type cells displayed similar responses to the envelope. The panel on the right shows however that, at smaller time scales, the ON-type cell fired preferentially near local maxima while the OFF-type cell fired preferentially near local minima of the carrier. **(B)** *Left:* Phase histograms wrt to the carrier for ON- (red) and OFF- (blue) type cells. ON- and OFF-type cells fire preferentially at opposite phases of the carrier waveform, which is expected. *Right:* Phase histograms wrt to the envelope for ON- (red) and OFF- (blue) time cells. It is seen that both displayed more similar distributions, which is consistent with previous results [1].

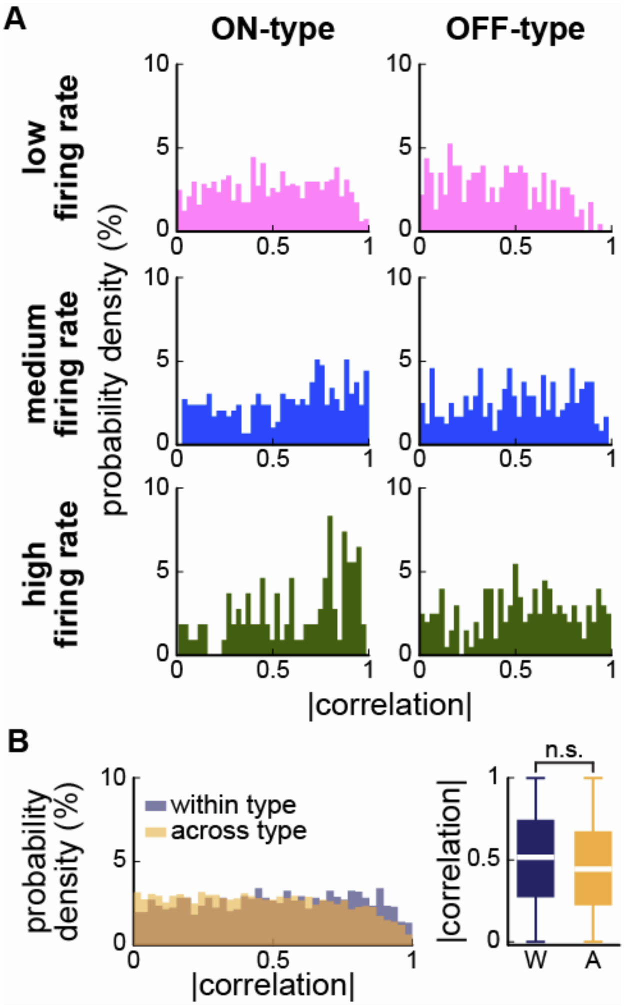

Supplementary Figure 2 related to Figure 2: *All ELL pyramidal cell types display similar levels of response heterogeneity to envelope stimuli.* **(A)** Distributions of absolute correlation coefficients obtained for all six cell types (ON and OFF low, medium, and high firing rate cells, respectively). These were not significantly different from one another (Friedman's test, df = 5, p = 0.56). **(B)** *Left:* Distribution of absolute correlation coefficients within cell type (purple) and across cell types (beige). *Right:* Whisker-box plots of absolute correlation coefficients obtained within cell type (purple) and across cell types (beige). No significant difference was found (two-sample KS test, D = 39, p = 0.51).

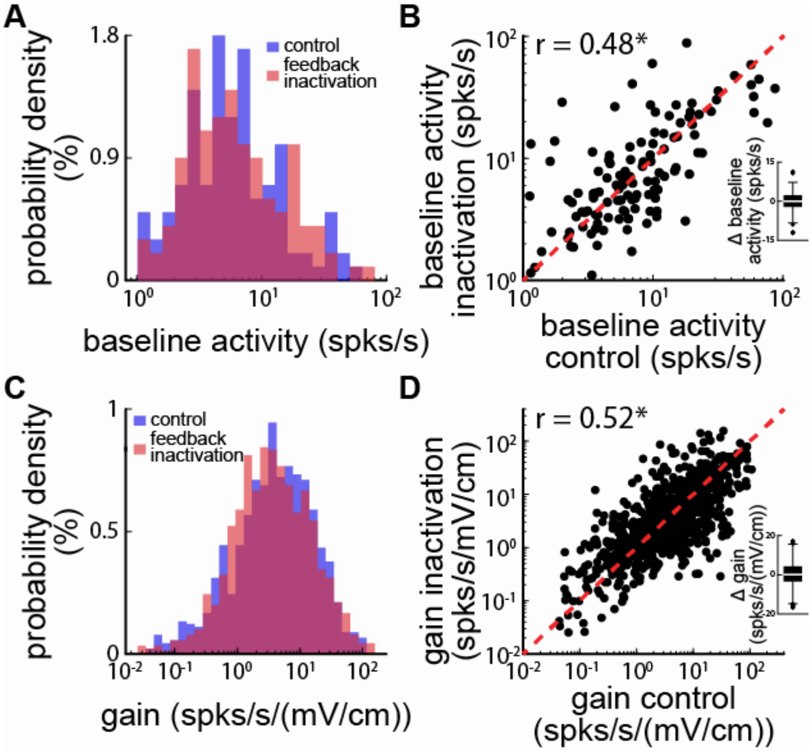

Supplementary Figure 3 related to Figure 2: *Both firing rate and stimulus sensitivity are not significantly altered by feedback inactivation.* **(A)** Probability densities for firing rate obtained before (blue) and after feedback inactivation. Both distributions were not significantly different from one another (KS test, p=0.93, N=19). **(B)** Plot showing baseline firing rate after feedback inactivation as a function of baseline firing rate before feedback inactivation for all neurons in our dataset. Datapoints were scattered across the identity line (red dashed line; r = 0.48, p = 1.2910^{-8}, N = 134). Inset: population-averaged change in baseline firing rate due to feedback inactivation was not significantly different from zero (p = 0.62, paired t-test, N = 134). **(C)** Probability densities for neural gain obtained before (blue) and after feedback inactivation. Both distributions were not significantly different from one another (two-sample KS test, p = 0.74, D = 29). **(D)** Gain after feedback inactivation as a function of gain before feedback inactivation for all neurons across conditions in our dataset. Datapoints were scattered across the identity line (red dashed line; r = 0.52, p = $2.44*10^{-61}$, N = 888). Inset: population-averaged change in gain due to feedback inactivation was not significantly different from zero (p = 0.25, paired t-test, N = 888).

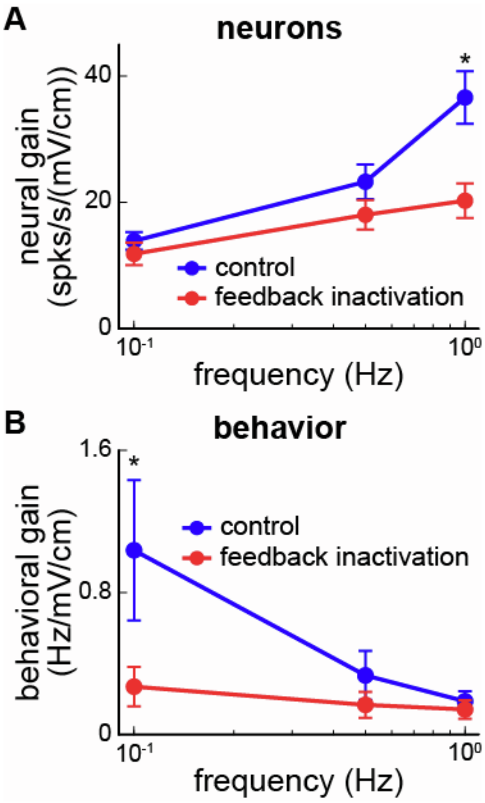

Supplementary Figure 4 related to Figure 2: *Effects of feedback inactivation on neural and behavioral sensitivity.* **(A)** Population-averaged neural gain as a function of envelope frequency before (blue) and after (red) feedback inactivation. We only included cells that clearly responded to the envelope (see Methods) for weak contrast in order to better compare with previous results. Overall, our results were similar to those obtained previously (compare with Fig. 4B of [2]). **(B)** Population-averaged behavioral gain as a function of envelope frequency before (blue) and after (red) feedback inactivation for low contrast. Overall, our results were similar to those obtained previously (compare with Fig. 4E of [2]). "*" Indicates statistical significance using a paired t-test at the p = 0.05 level ($N_{neurons}$ = 38; $N_{behavior}$ = 5).

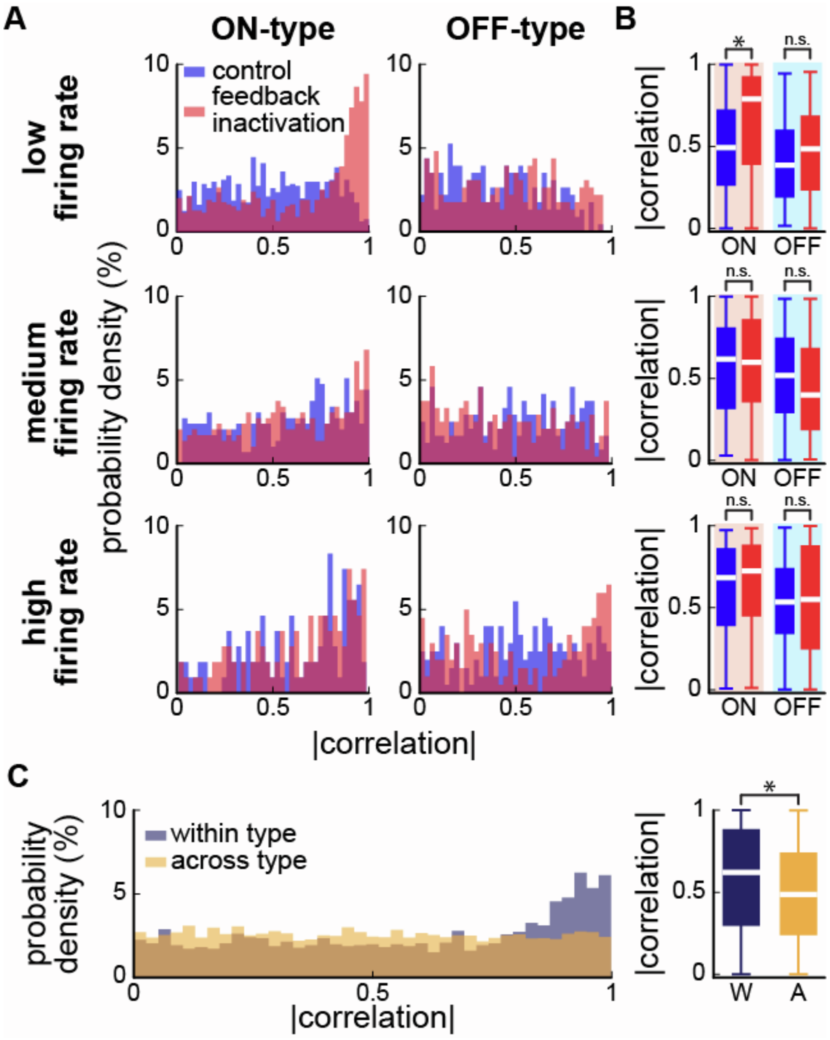

Supplementary Figure 5 related to Figure 2: *Feedback inactivation primarily reduces heterogeneity by reducing heterogeneity of low firing rate ON-type cells*. **(A)** *Left:* Distributions of absolute correlation coefficients obtained for all six cell types (ON and OFF low, medium, and high firing rate cells) before (blue) and after (red) feedback inactivation. **(B)** Whisker-box plots of absolute correlation coefficients obtained before (blue) and after (red) feedback inactivation. A significant difference was only seen for low firing rate ON-type cells (two-sample KS test, D = 39, p = $2.83*10^{-4}$) whereas no significant difference was seen for all other five cell types (OFF-type low firing rate: p = 0.51; ON-type medium firing rate: p = 0.51; OFF-type medium firing rate: p = 0.71; ON-type low firing rate: p = 0.98; OFF-type low firing rate: p = 0.71; two-sample KS tests, D = 39). **C)** *Left:* Distribution of absolute correlation coefficients within cell type (purple) and across cell types (beige) obtained after feedback inactivation. *Right:* Whisker-box plots of absolute correlation coefficients obtained within cell type (purple) and across cell types (beige). Correlation magnitude was significantly higher within cell type (two-sample KS test, D = 39, p = $1.04*10^{-6}$).

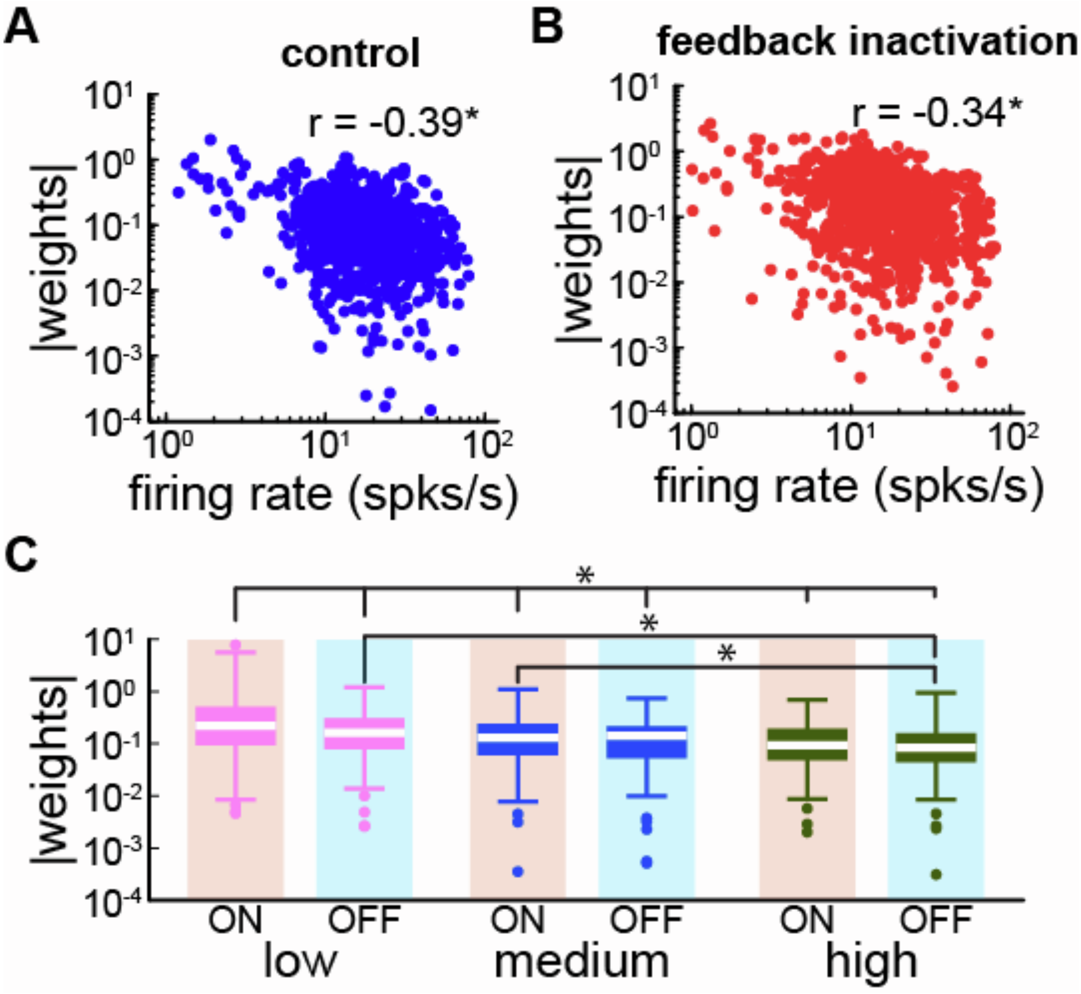

Supplementary Figure 6 related to Figure 3: *Weights associated with an optimal decoder are correlated with firing rate.* **(A)** Under control conditions (i.e., before feedback inactivation), weight magnitude was significantly negatively correlated with firing rate for the optimal decoder (i.e., weights are optimized to reconstruct the detailed timecourse of the stimulus) ($r = -0.39$, $p = 5.87*10^{-34}$, $N = 888$) before feedback inactivation. **(B)** Same as (A), but after feedback inactivation ($r = -0.34$, $p = 5.05*10^{-26}$, $N = 888$). **(C)** Whisker-box plots showing weight magnitude for all six cell types. “*” indicates statistical significance using a one-way ANOVA with Bonferroni correction ($F = 15.28$, $p = 1.95*10^{-14}$).

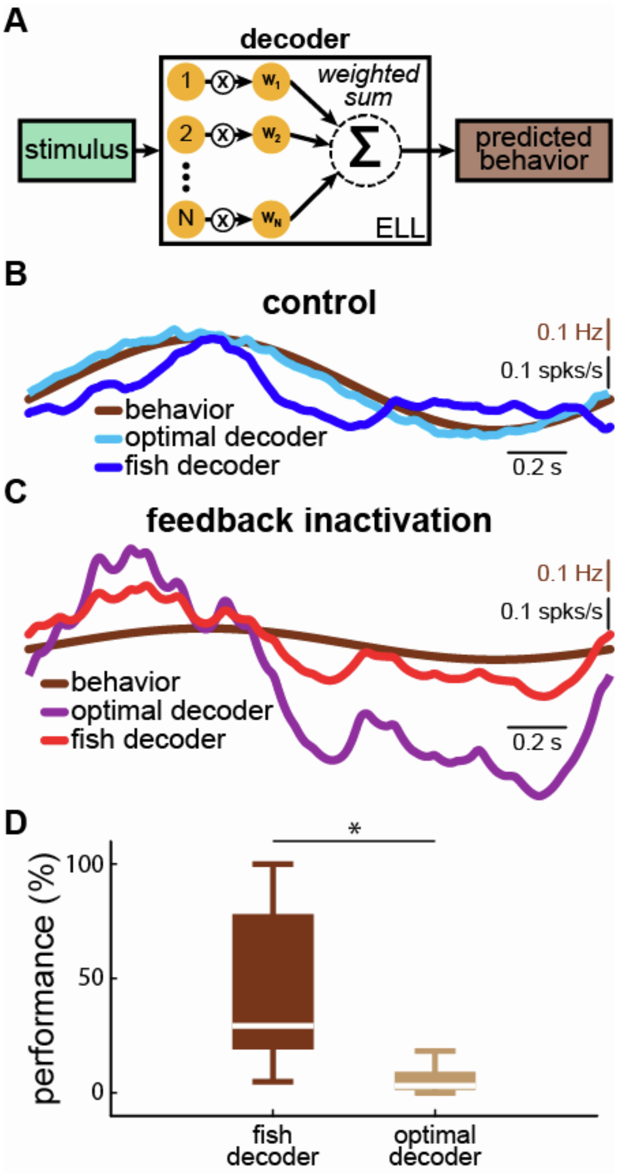

Supplementary Figure 7 related to Figures 3 and 4: *A global decoder is necessary to correctly predict changes in behavior due to feedback inactivation*. **(A)** Schematic showing the decoder. **(B)** Actual (brown) and predicted behavioral responses from a "local" decoder where the weights are independently optimized to minimize the mean square error for each condition separately (light blue) and from a global "fish" decoder where the weights are constant and chosen to minimize the mean square error across all conditions (blue) before feedback inactivation. It is seen that both decoders predict behavior. **(C)** Actual (brown) and predicted behavioral responses from the local (purple) and fish (red) decoder after feedback inactivation. It is seen that the global decoder gives a much better prediction than the local decoder, even though the weights were obtained exclusively under control conditions (i.e., before feedback inactivation). **(D)** Performance was significantly lower for the local decoder (light brown; right), than for the fish decoder (brown; left) ($p = 4.08*10^{-9}$, N = 40; paired t-test).

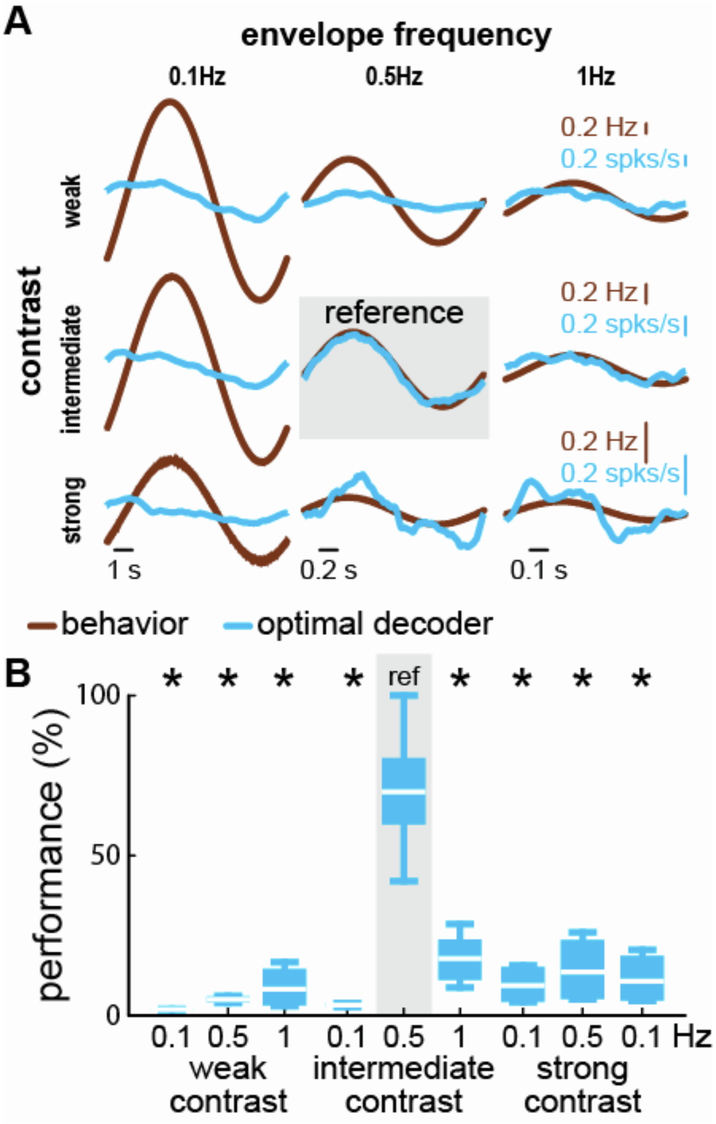

Supplementary Figure 8 related to Figures 3 and 4: *Using weights obtained for the optimal decoder for one condition perform poorly at predicting behavioral responses for all other conditions.* **(A)** Actual (brown) and predicted (blue) behavioral responses for all nine conditions for the optimal decoder with weigths obtained from the middle condition (i.e., the “reference”). **(B)** Decoding performance for all nine conditions. Overall, performance was significantly less for conditions other than the reference. “*” indicates statistical significance using a one-way ANOVA with Bonferroni correction ($F = 946.24$, $p < 0.0001$). Qualitatively similar results were obtained when taking all other conditions as reference.

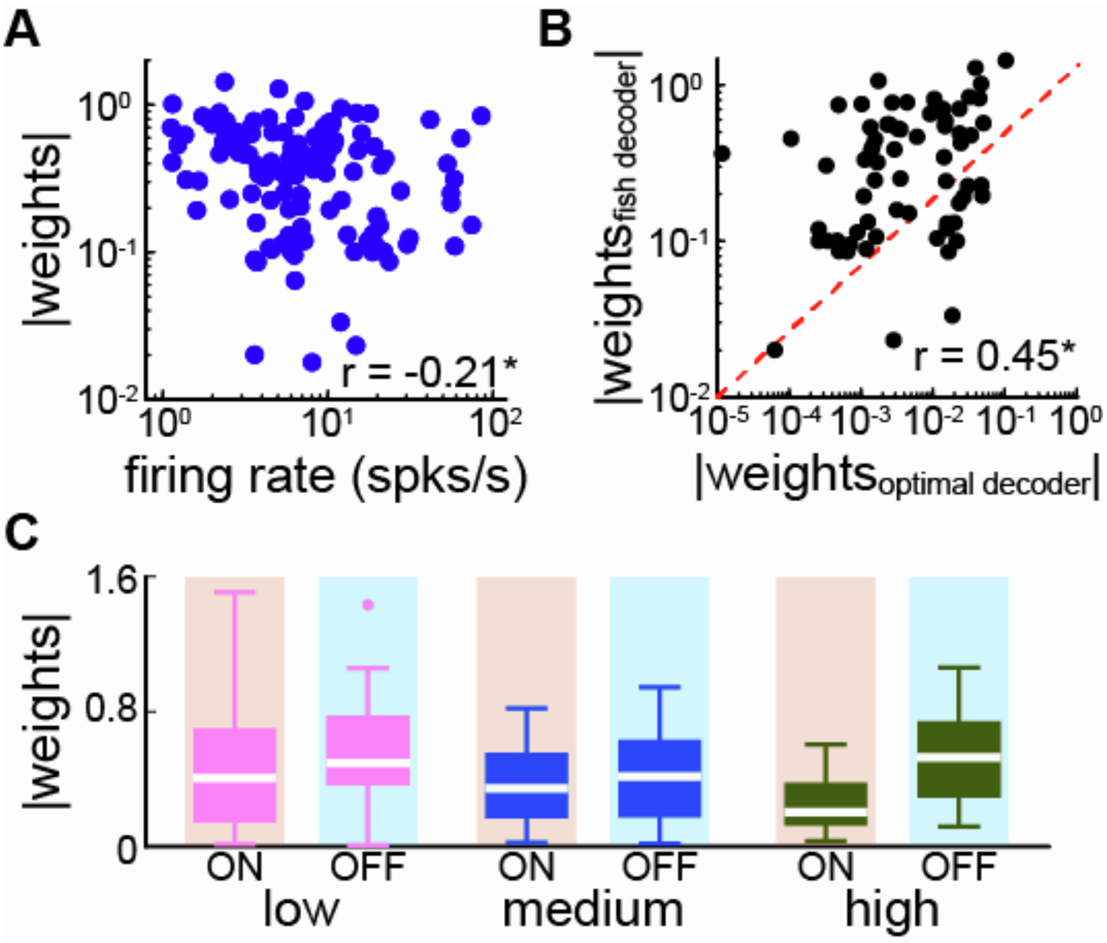

Supplementary Figure 9 related to Figures 3 and 4: *Weights associated with the fish decoder are correlated with firing rate and those associated with the optimal decoder.* **(A)** Weight magnitude was significantly negatively correlated with firing rate for the fish decoder (r = -0.21, p = 0.02, N = 134). **(B)** Weight magnitude associated with the fish decoder was significantly positively correlated with that associated with the optimal decoder (r = 0.45, p = 0.0004, N = 68). **(C)** Whisker-box plots showing weight magnitude for all six cell types. No significant differences were seen (one-way ANOVA with Bonferroni correction; F = 2.28, p = 0.05).

References:

1. Huang, C.G., and Chacron, M.J. (2016). Optimized Parallel Coding of Second-Order Stimulus Features by Heterogeneous Neural Populations. J Neurosci *36*, 9859-9872. 10.1523/JNEUROSCI.1433-16.2016.
2. Huang, C.G., Metzen, M.G., and Chacron, M.J. (2018). Feedback optimizes neural coding and perception of natural stimuli. Elife *7*, e38935. 10.7554/eLife.38935.
